# Two Preputial Gland-Secreted Pheromones Evoke Sexually Dimorphic Neural Pathways in the Mouse Vomeronasal System

**DOI:** 10.3389/fncel.2019.00455

**Published:** 2019-10-02

**Authors:** Qun Liu, Yaohua Zhang, Pan Wang, Xiao Guo, Yijun Wu, Jian-Xu Zhang, Liquan Huang

**Affiliations:** ^1^College of Life Sciences, Zhejiang University, Hangzhou, China; ^2^State Key Laboratory of Integrated Management of Pest Insects and Rodents in Agriculture, Institute of Zoology, Chinese Academy of Sciences, Beijing, China; ^3^Monell Chemical Senses Center, Philadelphia, PA, United States

**Keywords:** hexadecanol, hexadecyl acetate, vomeronasal organ, calcium imaging, c-Fos, neural circuits

## Abstract

Hexadecanol (16OH) and hexadecyl acetate (16Ac) are two pheromones secreted in a large quantity by mouse preputial glands and act on male and female mice differentially. Yet the underlying molecular and cellular mechanisms remain to be elucidated. In this study, we examined the activation of vomeronasal sensory neurons (VSNs) by these two pheromones and mapped the downstream neural circuits that process and relay their chemosignals. Using the calcium imaging method and immunohistochemistry, we found that a small number of VSNs were activated by 16OH, 16AC, or both in the male and female mice, most of which were located apically in the vomeronasal epithelium, and their numbers did not increase when the concentrations of 16OH and 16Ac were raised by 10,000-fold except that of female VSNs in response to 16OH. In the accessory olfactory bulb (AOB), the two pheromones evoked more c-Fos+ neurons in the anterior AOB (aAOB) than in the posterior AOB (pAOB); and the increases in the number of c-Fos+ neurons in both aAOB and pAOB were dose-dependent; and between sexes, the female AOB responded more strongly to 16OH than to 16Ac whereas the male AOB had the opposite response pattern. This sexual dimorphism was largely retained in the downstream brain regions, including the bed nucleus of the stria terminalis (BNST), the medial amygdaloid nucleus (MeA), the posteromedial cortical amygdaloid nucleus (PMCo), the medial preoptic area (MPA), and the ventromedial hypothalamic nucleus (VmH). Taken together, out data indicate that there is one V1r receptor each for 16OH, 16Ac, or both, and that activation of these receptors evokes sexually dimorphic neural circuits, directing different behavioral outputs and possibly modulating other pheromone-induced responses.

## Introduction

The term “pheromone” has been used to describe the chemicals used for intra-species communications (Karlson and Luscher, 1959). These chemicals can convey information about conspecific members’ social status, sexual maturation, and receptiveness, and can also trigger sexual behaviors, pregnancy block, inter-male or infant-directed aggression, and parental caring (Leinders-Zufall et al., 2000, 2004; Brennan and Zufall, 2006; Brechbühl et al., 2011; Liberles, 2014; Isogai et al., 2018; Mohrhardt et al., 2018). Since the first pheromones were identified in insects, many more have been found across the animal kingdom (Liberles, 2014). In mammals, rodents have been the best studied model animals, which emit pheromones through feces, urine, saliva, tear, and other bodily fluids that are produced by such glands as preputial and lacrimal glands (Novotny, 2003; Zhang et al., 2008; Brechbühl et al., 2011). However, the number of molecularly identified pheromones is still limited in mammals, including proteins and peptides, e.g., the major urinary proteins (MUPs) (Hurst et al., 2001), major histocompatibility complex (MHC) class I peptides (Sturm et al., 2013), exocrine gland-secreting peptides (ESPs) (Kimoto et al., 2005; Osakada et al., 2018), as well as more volatile compounds such as bile acids and steroids, e.g., 2,5-dimethylpyrazine, 3,4-dehydro-exo-brevicomin, 2,3-dehydro-exo-brevicomin, 2-heptanone, 6-hydroxy-6-methyl-3-heptanone (HMH), 2-sec-butyl-4,5-dihydrothiazole (SBT), α/β-farnesene, and sulfated sex hormones (Novotny et al., 1990, 1999; Brennan and Zufall, 2006; Haga-Yamanaka et al., 2014; Doyle et al., 2016). Naturally produced pheromones, however, are often mixtures, of which the ratios of these different components are also important to evoking the receivers’ behavioral responses. Identifying additional semiochemicals and their blends is indeed necessary to fully understand pheromone physiology.

The pheromones are detected mainly by the sensory neurons in the vomeronasal organs (VNO) that are located at the anterior bottom of murine nasal cavity. Unlike the olfactory epithelium of the main olfactory system (MOS), the vomeronasal epithelium (VSE) can be largely divided into apical and basal layers whereas the basal layer can be further divided into two sublayers (Munger et al., 2009; Leinders-Zufall et al., 2014; Liberles, 2014; Akiyoshi et al., 2018). And the vomeronasal sensory neurons (VSNs) in each layer express different pheromone receptors and signaling proteins: in mice, each apical VSN expresses one formyl peptide receptor (FPR) or 1 of the 239 potentially functional V1r receptors in a monoallelic fashion as well as the G-protein α-subunit Gα_i2_ whereas each basal VSN expresses two of 121 functional V2rs, both in monoallelic fashion, along with the G-protein α-subunit Gα_0_ (Zhang et al., 2004; Young and Trask, 2007; Riviere et al., 2009; Young et al., 2010; Akiyoshi et al., 2018). Among these 360 murine V1rs and V2rs, few have been deorphanized, largely due to the difficulty in heterologously expressing these receptors. Nevertheless, the information about the interactions between these receptors and their ligands is critical to revealing the subsequent pheromone information processing in the central neural circuits, and subsequently to determining the behavioral outcome (Keverne, 1999; Dulac and Torello, 2003; Halpern and Martınez-Marcos, 2003; Leinders-Zufall et al., 2004; Munger et al., 2009; Touhara and Vosshall, 2009; Liberles, 2014).

The anatomic division in the VSE is maintained when apical and basal VSNs project their axons to the anterior and posterior portions of the accessory olfactory bulb (AOB), respectively (Keverne, 1999; Halpern and Martınez-Marcos, 2003; Leinders-Zufall et al., 2004; Brennan and Zufall, 2006). Unlike in the main olfactory bulb (MOB) where the axons of olfactory sensory neurons expressing the same olfactory receptors converge onto one or two glomeruli, individual axons of VSNs can branch out in the AOB, innervating multiple glomeruli. Conversely, each glomerulus can be innervated by axons expressing different vomeronasal receptors, resulting in the glomeruli of variable sizes correlating to the numbers of afferent VSN fibers. This structural feature may indicate that chemosensory information is more intensively processed in the AOB than in the MOB before being relayed to the downstream circuits in the brain (Belluscio et al., 1999; Wagner et al., 2006).

In the mouse brain, two areas in the limbic system receive signals from mitral cells of the AOB: the bed nucleus of the stria terminalis (BNST) and the vomeronasal amygdala while the latter consists of the medial amygdaloid nucleus (MeA) and the posteromedial amygdaloid cortical nucleus (PMCo). Neurons from these nuclei further project to the hypothalamic nuclei including the medial preoptic area (MPA) and the ventromedial hypothalamus (VmH), which sends out the output signals directing physiological and behavioral responses (Halem et al., 1999; Halpern and Martınez-Marcos, 2003; Brennan and Zufall, 2006; Dulac and Wagner, 2006; Haga et al., 2010; Szymanski and Keller, 2014). Up till now, however, very few pheromones’ neural circuits have been mapped out (Dulac and Torello, 2003; Brennan and Zufall, 2006; Ishii et al., 2017; Wei et al., 2018). And even more complicated is that the accessory olfactory system (AOS) may also be plastic and can be modulated by learning and internal physiological states (Kaur et al., 2014; Xu et al., 2016). Thus, more thorough studies are needed to elucidate the AOS circuits beyond those merely eliciting the stereotyped responses.

Hexadecanol (16OH) and hexadecyl acetate (16Ac) are two pheromones initially identified from insects, and later found to be produced in a substantial quantity by murine preputial glands (McElfresh et al., 2001; Zhang et al., 2008). Male mice, which produce more of these two pheromones than females, show some attraction to them at low concentrations but display aversion to concentrated ones while female mice are indifferent to low concentrations but attractive to high concentrations of these two pheromones; and these sex-specific responses are VNO-dependent, and probably mediated by the AOS (Zhang et al., 2008). However, the exact molecular and cellular mechanisms underlying their detection in the VNO and information processing in the central circuits remain to be revealed. In this study, we utilized calcium imaging to identify the VSNs responsible for the 16OH and 16Ac detection, and c-Fos immunohistochemistry to identify activated neurons in the central nuclei including those in the AOB, BNST, PMCo, MeA, MPA, and VmH that make up the AOS neural circuits. Our data provide new insights into the underlying molecular and cellular mechanisms of the 16OH- and 16Ac-directed dimorphic behavioral outputs.

## Materials and Methods

### Animals

All the mice used in the experiments are 8–10 weeks old CD-1 mice. A total of 118 mice were used in this study: 5 male and 5 female mice were used for the VNO calcium imaging experiment; 24 male and 24 female mice for the pheromone-induced VNO pS6 immunofluorescence experiment, and 30 male and 30 female mice for the brain c-Fos immunohistochemistry experiment. Male mice were housed individually whereas females were housed in groups of four with food and water available *ad libitum.* Female mice were examined to determine their estrous stages and only estrous mice were used in this study. Before exposure to the pheromones, subjects were housed individually under a reversed 14/10 h light/dark photoperiod (lights on at 7:00 pm) with food and water available *ad libitum* in a clean room and the experiments were carried out in the morning hours. All experiments with the animals were approved by the Institutional Animal Care and Use Committees of both Zhejiang University (No. 14843) and Institute of Zoology, Chinese Academy of Sciences (IOZ 2015) and followed the NIH “Guide for the Care and Use of Laboratory Animals.”

### Calcium Imaging

#### VNO Slice Preparation

Animals were decapitated following anesthesia, and the mandible bone was cut off with a pair of scissors to remove the lower jaw. The ridged upper palate tissue was peeled off to expose the nasal cavity. The anterior and posterior ends of the nasal septum were cut to extract the VNO-containing portion, which was immediately transferred to the ice-cold oxygenated mouse artificial cerebro-spinal fluid (95% O_2_/5% CO_2_; mACSF containing 125 mM NaCl, 2.5 mM KCl, 1 mM MgCl_2_, 1.25 mM NaH_2_PO_4_, 25 mM NaHCO_3_, 2 mM CaCl_2_, 10 mM D-Glucose, pH 7.4) (Brechbühl et al., 2011; Ma et al., 2011). The cartilaginous capsule of the VNO was then removed to gain access to the luminal surface of the sensory epithelium. The dissected VNO was embedded in 3% low-melting agar and sliced coronally into 200 μm-thick sections with a vibratome at a speed of 0.5 mm/s and amplitude of 0.7 mm (Leinders-Zufall et al., 2000).

#### Calcium Imaging

A VNO tissue slice was loaded with 10 μM calcium-sensitive dye Fura-2-AM (F1201, Life Technology, United States) for 30–60 min. Calcium imaging was performed on a Nikon microscope equipped with 20×, 40× water immersion objectives to monitor the changes in intracellular Ca^2+^ concentrations over time. Cells were illuminated sequentially at 340/380 nm with a polychromator instrument and emission at 510 nm was recorded at a rate of 5 Hz. Changes in the intracellular emission ratio at 340 and 380 nm, i.e., ratio = F340/F380 nm, were monitored with Ratio Imaging software. Pheromones were delivered at a flow rate of 1 ml/min using a peristaltic pump. Near the end of each imaging session, 50 mM KCl in mACSF was applied to the VNO slice to check the viability and responsiveness of the cells and only the responsive VSNs were included in the *post hoc* data analyses. The interstimulus intervals were 4 min or longer to allow the recovery of the VSNs. Solutions of 16OH and 16Ac were prepared as stock solutions of 1 M by being dissolved in dimethyl sulfoxide (DMSO) and then diluted in mACSF to two concentrations used in this study: low (10 nM) and high (100 μM). Thus, these four solutions: 10 nM 16OH, 10 nM 16Ac, 100 μM 16OH, and 100 μM 16Ac were designated as L-16OH, L-16Ac, H-16OH, and H-16Ac, respectively (Leinders-Zufall et al., 2000; Isogai et al., 2011).

### Immunohistochemistry

#### Pheromone Treatment

To stimulate mice, 16OH and 16Ac stock solutions of 1 M were prepared in dichloromethane (DCM) and diluted to 100 μM in double distilled water for VNO experiment, or diluted to two concentrations: 10 nM and 100 μM in mineral oil for AOB and brain experiment; thus, the concentration of residual DCM in the pheromone solutions was 0.01% or less. Forty microliters of the pheromones or whole urine (positive control) or 0.01% DCM in double distilled water (negative control) for VNO staining, and pheromones or 0.01% DCM in mineral oil (negative control) for AOB and brain staining were added onto a piece of filter paper placed in a mould in the bedding of the subjects that had been previously housed individually for 2 days in a clean room. The treatment lasted for 45 min for the VNO experiments or 90 min for the AOB and brain experiments. Based on sex and treatment, the animals were divided into eight groups: male mice treated with urine (Male-urine), 0.01% DCM-containing double distilled water (Male-ddH_2_O), 16OH (Male-16OH), and 16Ac (Male-16Ac); and female mice treated with urine (Female-urine), 0.01% DCM-containing double distilled water (Female-ddH_2_O), 16OH (Female-16OH), and 16Ac (Female-16Ac) for the VNO experiments. And for the AOB and brain experiments, the animals were divided into 10 groups: male mice treated with 0.01% DCM-containing mineral oil (designated as M-control), L-16OH (M-L-16OH), L-16Ac (M-L-16Ac), H-16OH (M-H-16OH), or H-16Ac (M-H-16Ac); and female mice treated with 0.01% DCM-containing mineral oil (F-control), L-16OH (F-L-16OH), L-16Ac (F-L-16Ac), H-16OH (F-H-16OH), or H-16Ac (F-H-16Ac).

#### Tissue Preparation

After pheromone treatment, the mice were anesthetized with sodium pentobarbital (40 mg/kg) and transcardially perfused with PBS followed by 4% paraformaldehyde (PFA) in PBS. Then, the VNO, OB, and brain were dissected out, post-fixed overnight, and cryoprotected in 30% sucrose solution in PBS overnight. Coronal sections of the VNO (12 μm thick) and brain (40 μm thick) and sagittal sections of the OB (30 μm thick) were sliced and collected. The brain sections that contained the nuclei potentially involved in the processing of 16OH and 16Ac signals, including the BNST, MPA, anterior subdivision of the MeA, ventromedial hypothalamic nucleus (VmH), posteromedial cortical amygdaloid nucleus (PMCo), were collected (Supplementary Figure 1; Paxinos and Franklin, 2004). The intervals between sections from the brain and OB were 40 and 30 μm apart, respectively.

#### Double Immunostaining With Anti-pS6 and Anti-Gα_o_ Antibodies on the VNO Sections (Jiang et al., 2015)

The VNO sections were washed with PBS to remove the embedding medium and blocked by incubating with the blocking buffer (10% normal goat serum, 2% bovine serum albumin in 0.5% Triton X-100/PBS solution) for 1 h. The anti-Gα_o_ primary antibody (551, Medical and Biological Laboratories, Japan) was diluted at 1:2000 in the dilution buffer (2% normal goat serum, 2% BSA in 0.5% Triton X-100/PBS) and applied to the sections for 16 h, followed by the incubation with AF568-conjugated goat-anti-rabbit secondary antibody diluted at 1:500 (ab175471, Abcam, United Kingdom) for 1 h. The anti-pS6 antibody (4856S, Cell Signaling Technology, Japan) was conjugated with AF488 using Zenon Rabbit lgG Labeling Kit (Z25302, Molecular Probes, United States) and applied to the sections for 1–2 h. Finally, the sections were washed with 1× PBS and covered with the mounting medium containing DAPI.

#### c-Fos Immunohistochemistry

Tissue sections were washed with PBS and incubated in 3% hydrogen peroxide in PBS for 30 min to eliminate background signals. After incubation with the blocking buffer for 1 h, tissue sections were incubated with the anti-c-Fos primary antibody (ab190289, Abcam, United Kingdom) diluted at 1:2000 for 16 h at 4°C, and then with the goat-anti-rabbit secondary antibody (BA-1000, Vector Laboratories, United States) diluted at 1:300 for 1 h, and finally with the avidin–biotin–peroxidase complex solution (PK-6100, ABC Kit, Vector Laboratories, United States) for 40 min. The sections were reacted with DAB Substrate Kit (SK-4100, Vector Laboratories, United States) for 2–3 min (Halem et al., 1999; Honda et al., 2008; Fabianová et al., 2014), and imaged with a Leica panoramic tissue cell scanning microscope.

#### Double Immunostaining With Anti-c-Fos and Anti-Gα_i2_ Antibodies

Following the c-Fos immunohistochemistry described above, the sections were blocked again in the blocking buffer, and then incubated with the anti-Gα_i2_ primary antibody (sc-13534, Santa Cruz, United States) at 1:500 dilution for 16 h at 4°C, followed by incubation with the AF488-conjugated goat-anti-mouse secondary antibody (A32723, Life Technologies, United States) at 1:2000 dilution for 1 h. Finally, the sections were washed with 1× PBS and covered with the mounting medium containing DAPI.

#### Quantification of c-Fos+ Cells

The numbers of the c-Fos expressing cells in the different regions of the AOB and brain were counted using the Image J software. The experimenter was blind to the treatments. And the thresholds of c-Fos signal intensity and the cell size were set to cover all identifiable c-Fos+ neurons in the image. The numbers of c-Fos+ cells in the glomerular layer (GL), mitral/tufted cell (MTC) layer, and the granule cell (GC) layer were summed up as the total numbers of the activated cells in the anterior and posterior AOB halves. For the brain regions, c-Fos+ cells from five sections located at the same distance of bregma were counted for a particular brain region (Supplementary Figure 2) and the mean value was calculated as the final number of activated neurons from that region of an animal (Paxinos and Franklin, 2004).

### Statistical Analysis

Calcium imaging data were expressed as the mean ± s.e.m. The data distribution and homogeneity of variances were checked. The differences between groups with different sexes, pheromones, and concentrations were assessed by two-way ANOVA with sex as between-group factor and treatment as within-group factor. If significant interaction was found, a *post hoc* analysis with Bonferroni’s multiple comparison tests was performed to identify significant differences with PASW Statistics 18.0 software. c-Fos immunohistochemistry data were also expressed as the mean ± s.e.m, and checked for distribution and homogeneity of variances. Two-way ANOVA was performed with both sex and treatment as between-group factors. After the significant interaction was found, similar *post hoc* analyses with Bonferroni’s multiple comparison tests were carried out as with the calcium imaging data using PASW Statistics 18.0 software.

## Results

### Hexadecanol and Hexadecyl Acetate Can Activate the Vomeronasal Sensory Neurons of Male and Female Mice

**FIGURE 1:**
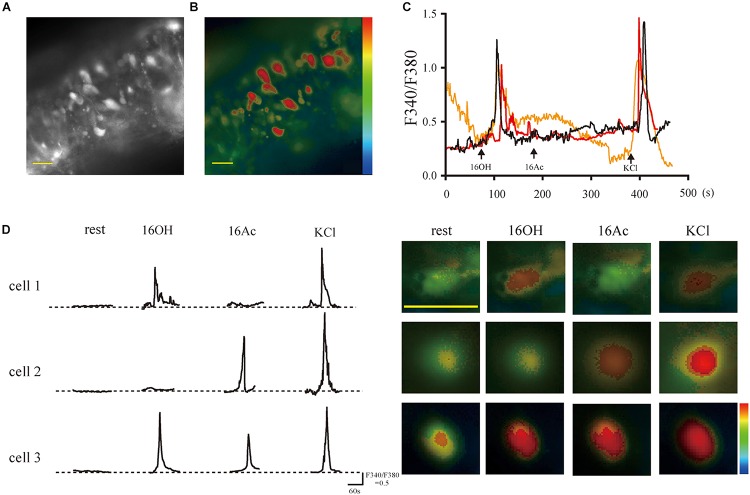
The pheromones evoke responses of intracellular Ca^2+^ elevation from single vomeronasal sensory neurons. **(A,B)** Fluorescence images on gray and pseudocolor scales, respectively, acquired from a VNO slice loaded with fura-2-AM. The somata of the VSNs contained most of the calcium-sensitive dye. **(C)** Traces of three VSNs responsive to 100 μM 16OH and KCl (50 mM) but not to 100 μM 16Ac over a period of 8 min. **(D) Left:** Response profiles of three representative VSNs: cell 1 responded to 16OH alone, cell 2 to 16Ac alone, and cell 3 to both; **Right:** the corresponding Fura-2 ratio images of the three VSNs. KCl (50 mM) was used as a positive control. Scale bar: 20 μm.

To determine at the cellular level whether hexadecanol (16OH) and hexadecyl acetate (16Ac) can indeed function as pheromones on mice, we investigated VSN responses to these two compounds using calcium imaging on the vomeronasal sensory epithelium slices. The VSNs were loaded with the calcium-sensitive dye Fura 2-AM and stimulated sequentially with the two pheromones (Figures 1A,B). The results showed that increases in the intracellular calcium concentrations were observed in some VSNs in response to 100 μM 16OH and 100 μM 16Ac. Based on their responsiveness profiles, these VSNs could be classified into four types: those responsive to 16OH alone, to 16Ac alone, to both, or neither (Figures 1C,D).

**FIGURE 2:**
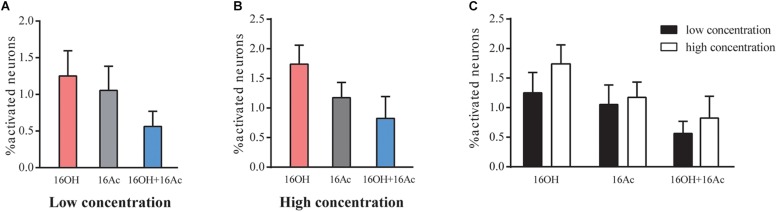
Male VSNs respond similarly to 16OH and 16OHAc at both low and high concentrations. **(A)** Comparison of the percentages of male VSNs responsive to 16OH and 16Ac at a low concentration (10 nM). Out of 1175 cells examined, 14 responded to 16OH, 11 to 16Ac, 7 to both 16OH and 16Ac; and no statistically significant difference was found among them (M-L-16OH vs. M-L-16Ac, *t* = 0.383, *p* = 1.000; M-L-16OH vs. M-L-16OH+M-L-16Ac, *t* = 1.338, *p* = 1.000; M-L-16Ac vs. M-L-16OH+M-L-16Ac, *t* = 0.956, *p* = 1.000). **(B)** Comparison of the percentages of male VSNs responsive to 16OH and 16Ac at a high concentration (100 μM). Out of 988 cells examined, 17 responded to 16OH, 11 to 16Ac, 8 to both 16OH and 16Ac; and no statistically significant difference was found among them (M-H-16OH vs. M-H-16Ac, *t* = 1.1, *p* = 1.000; M-H-16OH vs. M-H-16OH+M-H-16Ac, *t* = 1.784, *p* = 1.000; M-H-16Ac vs. M-H-16OH+M-H-16Ac, *t* = 0.683, *p* = 1.000). **(C)** Comparison analysis of the male VSNs responsive to the low and high concentrations of the pheromones. No significant difference was found between the responses to the low and high concentrations of the same pheromones in male mice (M-H-16OH vs. M-L-16OH, *t* = 0.953, *p* = 1.000; M-H-16Ac vs. M-L-16Ac, *t* = 0.236, *p* = 1.000; M-H-16OH+M-H-16Ac vs. M-L-16OH+M-L-16Ac, *t* = 0.508, *p* = 1.000). All values are expressed as mean ± s.e.m.

**FIGURE 3:**
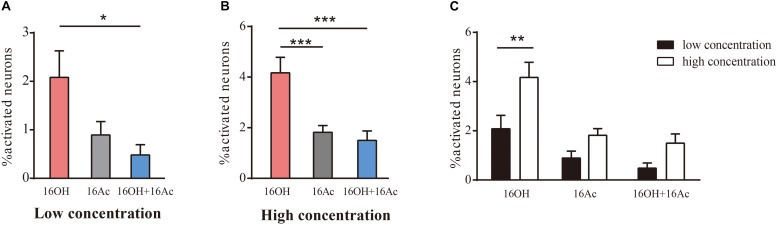
Female VSNs respond differently to 16OH and 16Ac. **(A)** Comparison of the percentages of female VSNs responsive to 16OH and 16Ac at 10 nM. Out of 1168 cells examined, 23 responded to L-16OH, 10 to L-16Ac, and 6 to both. Significantly more cells responded to L-16OH than to both; and no significant differences in the numbers of cells responsive to L-16OH versus to L-16Ac or to L-16Ac versus to both were found (F-L-16OH vs. F-L-16Ac, *t* = 2.311, *p* = 0.341; F-L-16OH vs. F-L-16OH + F-L-16Ac, *t* = 3.116, *p* = 0.035; F-L-16Ac vs. F-L-16OH + F-L-16Ac, *t* = 0.805, *p* = 1.000). **(B)** Comparison of the percentages of female VSNs responsive to 16OH and 16Ac at 100 μM. Out of 964 cells examined, 41 responded to H-16OH, 17 to H-16Ac, and 16 to both. Cells responsive to H-16OH were significantly more than those to H-16Ac or both but no significant difference was found between cells responsive to H-16Ac versus both (F-H-16OH vs. F-H-16Ac, *t* = 4.573, *p* = 0.000; F-H-16OH vs. F-H-16OH + F-H-16Ac, *t* = 5.196, *p* = 0.000; F-H-16Ac vs. F-H-16OH + F-H-16Ac, *t* = 0.623, *p* = 1.000). **(C)** Comparative analysis of the female VSNs responsive to the same pheromones at different concentrations. More female VSNs responded to H-16OH than to L-16OH whereas no significant differences were found between cell numbers responsive to H- versus to L-16Ac or between those to both H-16OH and H-16Ac versus both L-16OH and L-16Ac (F-H-16OH vs. F-L-16OH, *t* = 4.062, *p* = 0.001; F-H-16Ac vs. F-L-16Ac, *t* = 1.800, *p* = 1.000; F-H-16OH + F-H-16Ac vs. F-L-16OH + F-L-16Ac, *t* = 1.982, *p* = 0.751). All values are expressed as mean ± s.e.m. ^∗^*p* < 0.05, ^∗∗^*p* < 0.01, ^∗∗∗^*p* < 0.001.

From 5 male mice, out of 1175 VSNs examined, 14 (1.19%), 11 (0.93%), and 7 (0.6%) of VSNs responded to L-16OH, L-16Ac, or both, respectively (Figure 2A) whereas out of 988 VSNs examined, 17 (1.7%), 11 (1.1%), and 8 (0.81%) responded to H-16OH, H-16Ac, or both, respectively (Figure 2B). On the other hand, from 5 female mice, out of 1168 VSNs examined, 23 (1.97%), 10 (0.86%), and 6 (0.51%) responded to L-16OH, L-16Ac, or both, respectively (Figure 3A) whereas out of 964 VSNs examined, 41 (4.25%) responded to H-16OH, 17 (1.76%) to H-16Ac, and 16 (1.66%) to both, respectively (Figure 3B). Two-way ANOVA analysis with sex as a between-group factor and pheromone treatment as a within-group factor found that the effects of interaction (*F*_(__5,90__)_ = 3.3, *p* = 0.0088), sex (*F*_(__1,18__)_ = 11.8, *p* = 0.0029), and pheromone treatment (*F*_(__5,90__)_ = 10.56, *p* < 0.0001) were all significant. A *post hoc* analysis with Bonferroni’s correction showed that no statistically significant difference was found among the numbers of cells responsive to L-16OH, L-16Ac, or both (Figure 2A), or among the numbers of cells responsive to H-16OH, H-16Ac, or both (Figure 2B), neither was found between the numbers of cells responsive to L- versus H-16OH, or L- versus H-16Ac in the male mice (Figure 2C).

In the female mice, however, significantly more VSNs responded to L-16OH than to both, whereas no significant differences in the numbers of the VSNs responsive to L-16OH versus to L-16Ac or to L-16Ac versus to both L-16OH and L-16Ac were found (Figure 3A). When the high concentration was applied, significantly more VSNs responded to H-16OH than to H-16Ac or to both, whereas cells responsive to H-16Ac were not significantly more than those to both (Figure 3B). Further analysis showed that H-16OH activated more female VSNs than L-16OH, but the numbers of the cells responsive to 16Ac alone or to both were unaffected by their concentrations (Figure 3C).

**FIGURE 4:**
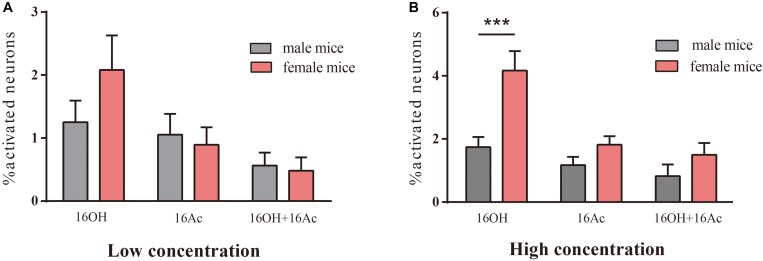
The differences in responsivity between male and female mice. **(A)** No significant differences were found between the numbers of male versus female VSNs responsive to L-16OH, L-16Ac, or both at a low concentration (10 nM) (M-L-16OH vs. F-L-16OH, *t* = 1.618, *p* = 0.109; M-L-16Ac vs. F-L-16Ac, *t* = 0.310, *p* = 0.757; M-L-16OH + M-L-16Ac vs. F-L-16OH + F-L-16Ac, *t* = 0.160, *p* = 0.873). **(B)** Significantly more female VSNs responded to H-16OH than male VSNs whereas no significant differences were found between the numbers of male and female responsive cells to H-16Ac or to both (100 μM) (M-H-16OH vs. F-H-16OH, *t* = 4.726, *p* = 0.000; M-H-16Ac vs. F-H-16Ac, *t* = 1.254, *p* = 0.213; M-H-16OH + M-H-16Ac vs. F-H-16OH + F-H-16Ac, *t* = 1.314, *p* = 0.192). All values are expressed as mean ± s.e.m. ^∗∗∗^*p* < 0.001.

Comparative analysis of the VSN responsiveness between sexes indicated that no significant differences were found between male and female VSNs in response to L-16OH alone, L- or H-16Ac alone, both L-16OH and L-16Ac, or both H-16OH and H-16Ac except that significantly more female VSNs than male VSNs responded to H-16OH (Figure 4).

### The Activated VSNs Are Mostly Located in the Apical Vomeronasal Sensory Epithelium

**FIGURE 5:**
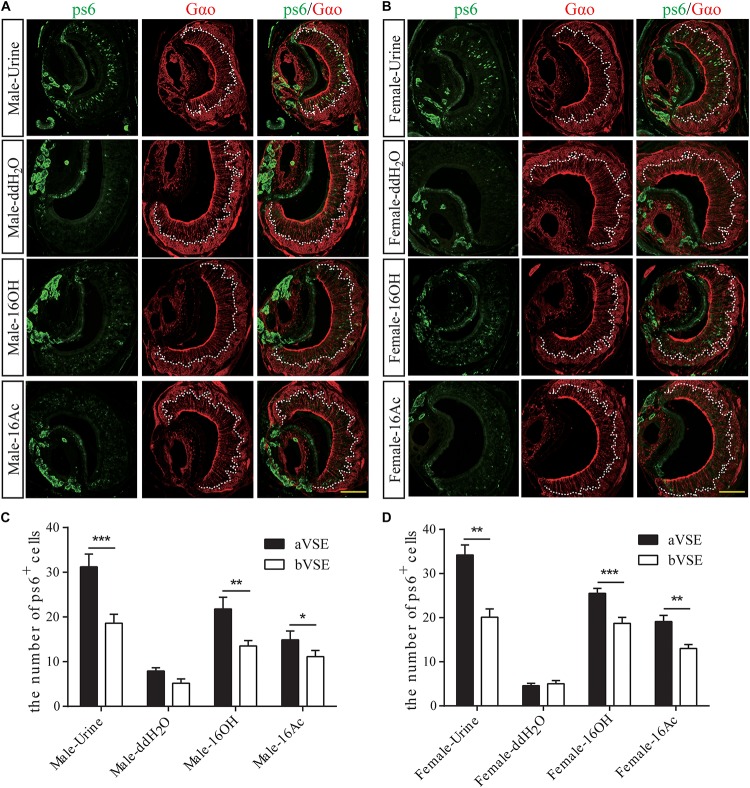
Most responsive neurons are apical VSNs. **(A,B)** Images of anti-pS6 and anti-Gα_o_ double immunostaining on male and female VNO sections, respectively, following the exposure to 100 μM 16OH, 100 μM 16Ac, urine (positive control), or double distilled water containing 0.01% DCM (ddH_2_O, negative control). **(C,D)** Comparative analysis of the double immunostaining data from both the male and female VNO sections, respectively, showed that significantly more pS6-positive neurons were located in the apical VSE (aVSE) than in the basal VSE (bVSE) upon stimulation of urine, 16OH, or 16Ac (aVSE vs. bVSE: male-urine, *t* = 6.350, *p* = 0.000; male-16OH, *t* = 3.275, *p* = 0.010; male-16Ac, *t* = 2.604, *p* = 0.029; female-urine, *t* = 4.462, *p* = 0.002; female-16OH, *t* = 6.464, *p* = 0.000; female-16Ac, *t* = 4.620, *p* = 0.001). All values are expressed as mean ± s.e.m. ^∗^*p* < 0.05, ^∗∗^*p* < 0.01, ^∗∗∗^*p* < 0.001.

To determine which type of the VSNs responsive to the two pheromones, we used an antibody to the phosphorylated small ribosomal protein 6 (pS6) to identify activated VSNs and another antibody to Gαo to distinguish the apical layer from the basal layer of the vomeronasal sensory epithelium (Figures 5A,B; Jiang et al., 2015). The results show that significantly more pS6-expressing VSNs in both male and female mice were located in the apical VSE than in the basal VSE in response to 16OH and 16Ac, which was similar to the pattern evoked by mouse urine, whereas the numbers of the activated neurons between the aVSE and bVSE of the control mice exposed to double distilled water (ddH_2_O) were not significantly different (Figures 5C,D).

### Projection of 16OH and 16Ac Signals to the Accessory Olfactory Bulb

**FIGURE 6:**
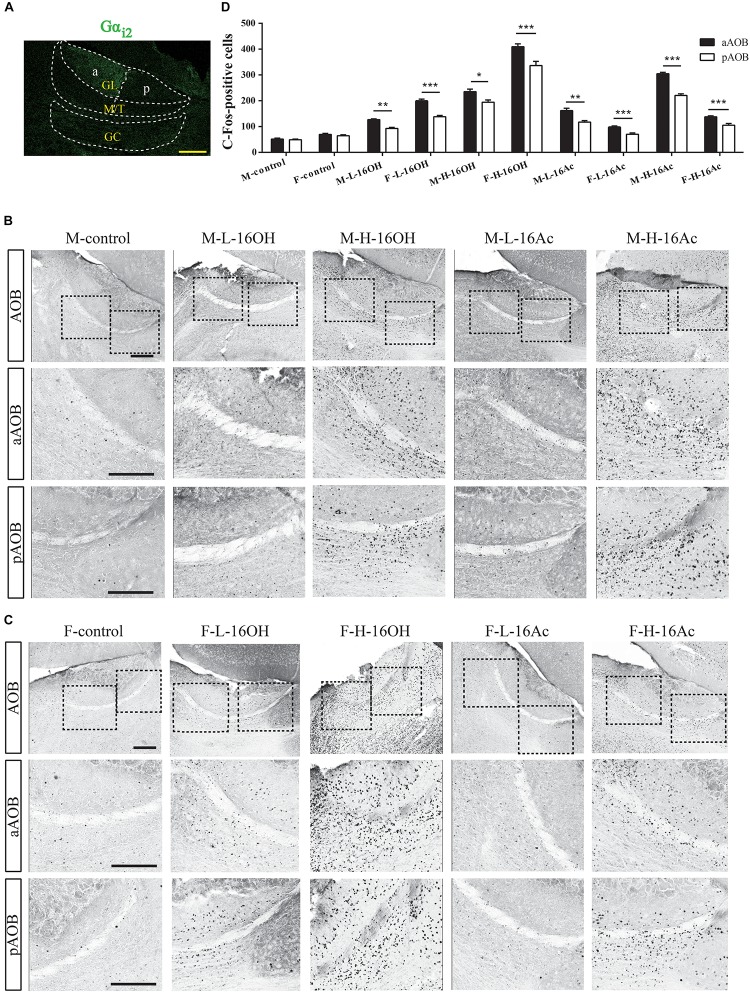
c-Fos+ neurons are found in mouse aAOB and pAOB after exposure to 16OH and 16Ac. **(A)** Gα_i2_ immunostaining was used to identify the anterior and posterior halves of the AOB (aAOB and pAOB); dotted lines denoted the mitral and tufted cell layer (M/T) and granule cell layer (GC). **(B)** c-Fos-immunostaining images of male AOB sections stimulated by L-16OH (M-L-16OH), H-16OH (M-H-16OH), L-16Ac (M-L-16Ac), H-16Ac (M-H-16Ac) as well as mineral oil containing 0.01% DCM (M-control): **Top** row, the entire AOB; **Middle** and **Bottom** rows, regions of the aAOB and pAOB at a higher magnification, respectively. **(C)** c-Fos-immunostaining images of the female AOB sections stimulated by L-16OH (F-L-16OH), H-16OH (F-H-16OH), L-16Ac (F-L-16Ac), H-16Ac (F-H-16Ac) as well as 0.01% DCM-containing mineral oil (F-control): **Top** row, the entire AOB; **Middle** and **Bottom** rows, aAOB and pAOB regions at a higher magnification, respectively. Scale bar, 200 μm. **(D)** Comparative analysis of the numbers of c-Fos+ neurons in the aAOB versus pAOB in response to both low and high 16OH and 16Ac indicated that aAOB displayed significantly more c-Fos+ neurons than pAOB in responses to any one of these four stimuli (aAOB vs. pAOB: M-L-16OH, *t* = 6.680, *p* = 0.001; M-H-16OH, *t* = 5.566, *p* = 0.003; F-L-16OH, *t* = 9.868, *p* = 0.000; F-H-16OH, *t* = 9.399, *p* = 0.000; M-L-16Ac, *t* = 6.526, *p* = 0.001; M-H-16Ac, *t* = 10.043, *p* = 0.000; F-L-16Ac, *t* = 14.029, *p* = 0.000; F-H-16Ac, *t* = 7.243, *p* = 0.000). *N* = 6 for each group. All values are expressed as mean ± s.e.m. ^∗^*p* < 0.05, ^∗∗^*p* < 0.01, ^∗∗∗^*p* < 0.001.

**FIGURE 7:**
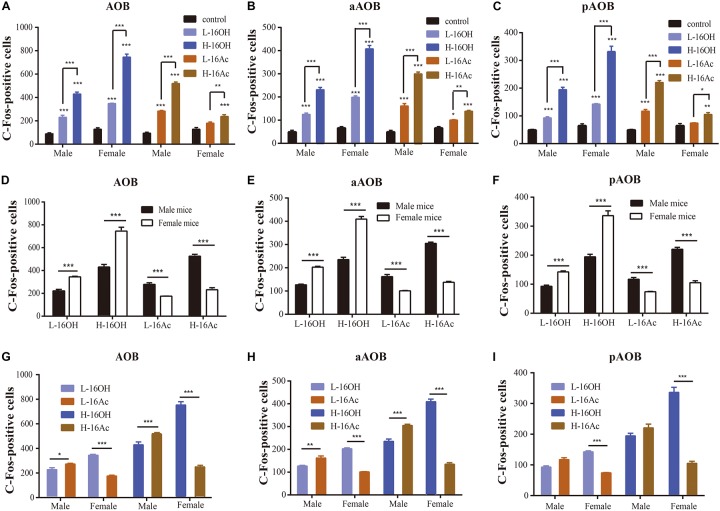
Quantitative analysis of c-Fos+ AOB neurons of the male and female mice after exposure to 16OH and 16Ac. **(A)** Significantly more activated neurons were found in both male and female aAOB and pAOB following stimulation by all these four stimuli: low or high 16OH or 16Ac in comparison with the control samples except the L-16Ac in female mice (M-L-16OH vs. M-control, *t* = 6.780, *p* = 0.000; M-H-16OH vs. M-control, *t* = 18.717, *p* = 0.000; M-L-16Ac vs. M-control, *t* = 10.148, *p* = 0.000; M-H-16Ac vs. M-control, *t* = 24.142, *p* = 0.000; F-L-16OH vs. F-control, *t* = 11.996, *p* = 0.000; F-H-16OH vs. F-control, *t* = 34.776, *p* = 0.000; F-L-16Ac vs. F-control, *t* = 2.364, *p* = 0.220; F-H-16Ac vs. F-control, *t* = 6.200, *p* = 0.000). H-16OH or H-16Ac evoked more c-Fos+ AOB neurons than the corresponding L-16OH and L-16Ac, respectively, in the mice of the same sex (M-H-16OH vs. M-L-16OH, *t* = 11.937, *p* = 0.000; M-H-16Ac vs. M-L-16Ac, *t* = 13.994, *p* = 0.000; F-H-16OH vs. F-L-16OH, *t* = 22.780, *p* = 0.000; F-H-16Ac vs. F-L-16Ac, *t* = 3.836, *p* = 0.004). **(B)** Comparative analysis of the c-Fos+ cells in the aAOB of the same sex exposed to the same pheromone at either the low or high concentration. Significantly more activated neurons were found in both male and female aAOB following stimulation by all these four stimuli: low or high 16OH or 16Ac in comparison with the control samples whereas H-16OH or H-16Ac evoked more c-Fos+ aAOB neurons than the corresponding L-16OH and L-16Ac, respectively, in the mice of the same sex (M-L-16OH vs. M-control, *t* = 8.302, *p* = 0.000; M-H-16OH vs. M-control, *t* = 20.212, *p* = 0.000; M-H-16OH vs. M-L-16OH, *t* = 11.910, *p* = 0.000; M-L-16Ac vs. M-control, *t* = 12.126, *p* = 0.000; M-H-16Ac vs. M-control, *t* = 27.836, *p* = 0.000; M-H-16Ac vs. M-L-16Ac, *t* = 10.078, *p* = 0.000; F-L-16OH vs. F-control, *t* = 14.637, *p* = 0.000; F-H-16OH vs. F-control, *t* = 37.370, *p* = 0.000; F-H-16OH vs. F-L-16OH, *t* = 22.733, *p* = 0.000; F-L-16Ac vs. F-control, *t* = 3.499, *p* = 0.010; F-H-16Ac vs. F-control, *t* = 7.529, *p* = 0.000; F-H-16Ac vs. F-L-16Ac, *t* = 4.030, *p* = 0.002). **(C)** Comparative analysis of the c-Fos+ cells in the pAOB of the same sex mice exposed to the same pheromone at the either low or high concentration (M-L-16OH vs. M-control, *t* = 4.266, *p* = 0.000; M-H-16OH vs. M-control, *t* = 14.180, *p* = 0.000; M-H-16OH vs. M-L-16OH, *t* = 9.915, *p* = 0.000; M-L-16Ac vs. M-control, *t* = 6.651, *p* = 0.000; M-H-16Ac vs. M-control, *t* = 16.729, *p* = 0.000; M-H-16Ac vs. M-L-16Ac, *t* = 10.078, *p* = 0.000; F-L-16OH vs. F-control, *t* = 7.596, *p* = 0.000; F-H-16OH vs. F-control, *t* = 26.515, *p* = 0.000; F-H-16OH vs. F-L-16OH, *t* = 18.919, *p* = 0.000; F-L-16Ac vs. F-control, *t* = 0.950, *p* = 1.000; F-H-16Ac vs. F-control, *t* = 3.639, *p* = 0.002; F-H-16Ac vs. F-L-16Ac, *t* = 3.007, *p* = 0.041). **(D)** Comparative analysis of c-Fos+ neurons in the AOB of males versus females stimulated by 16OH or 16Ac at the low or high concentration (F-L-16OH vs. M-L-16OH, *t* = 7.119, *p* = 0.000; F-H-16OH vs. M-H-16OH, *t* = 17.963, *p* = 0.000; M-L-16Ac vs. F-L-16Ac, *t* = 5.881, *p* = 0.000; M-H-16Ac vs. F-H-16Ac, *t* = 16.039, *p* = 0.000). **(E,F)** Comparative analysis of c-Fos+ neurons to 16OH and 16Ac between male and female aAOB and pAOB, respectively. Female aAOB and pAOB responded more strongly to L- or H-16OH than male aAOB and pAOB, respectively, whereas male aAOB and pAOB did more strongly to L- or H-16Ac than female aAOB and pAOB, respectively (aAOB: F-L-16OH vs. M-L-16OH, *t* = 8.307, *p* = 0.000; F-H-16OH vs. M-H-16OH, *t* = 19.130, *p* = 0.000; M-L-16Ac vs. F-L-16Ac, *t* = 6.655, *p* = 0.000; M-H-16Ac vs. F-H-16Ac, *t* = 18.335, *p* = 0.000; pAOB: F-L-16OH vs. M-L-16OH, *t* = 4.844, *p* = 0.000; F-H-16OH vs. M-H-16OH, *t* = 13.849, *p* = 0.000; M-L-16Ac vs. F-L-16Ac, *t* = 4.187, *p* = 0.000; M-H-16Ac vs. F-H-16Ac, *t* = 11.258, *p* = 0.000). **(G–I)** Comparison of the efficacy of 16OH versus 16Ac on evoking the c-Fos response in the AOB. 16OH seemed to be more effective on the female AOB, aAOB, or pAOB than on the male counterparts while 16Ac was more effective on male AOB, aAOB, or pAOB than on the female counterparts (AOB: M-L-16Ac vs. M-L-16OH, *t* = 3.368, *p* = 0.015; M-H-16Ac vs. M-H-16OH, *t* = 5.426, *p* = 0.000; F-L-16OH vs. F-L-16Ac, *t* = 9.632, *p* = 0.000; F-H-16OH vs. F-H-16Ac, *t* = 28.576, *p* = 0.000; aAOB: M-L-16Ac vs. M-L-16OH, *t* = 3.825, *p* = 0.004; M-H-16Ac vs. M-H-16OH, *t* = 7.624, *p* = 0.000; F-L-16OH vs. F-L-16Ac, *t* = 11.138, *p* = 0.000; F-H-16OH vs. F-H-16Ac, *t* = 29.841, *p* = 0.000; pAOB: M-L-16Ac vs. M-L-16OH, *t* = 2.386, *p* = 0.209; M-H-16Ac vs. M-H-16OH, *t* = 2.548, *p* = 0.139; F-L-16OH vs. F-L-16Ac, *t* = 6.646, *p* = 0.000; F-H-16OH vs. F-H-16Ac, *t* = 22.559, *p* = 0.000). *N* = 6 for each group. All values are expressed as mean ± s.e.m. ^∗^*p* < 0.05, ^∗∗^*p* < 0.01, ^∗∗∗^*p* < 0.001.

To determine the projection of 16OH and 16Ac signals from the VNO to AOB, we used c-Fos and Gαi2 immunostaining to identify and locate the activated neurons to the anterior or posterior half of the AOB (Figure 6). The numbers of c-Fos+ neurons in the AOB were significantly greater in both male and female mice exposed to both low and high 16OH and 16Ac than that in the control mice exposed to 0.01% DCM-containing mineral oil (Figure 6 and Table 1). In comparison of the c-Fos expression between the two AOB halves, we found that the aAOB had more c-Fos+ neurons than the pAOB in both male and female mice in response to low and high 16OH or 16Ac (Figure 6D and Table 1). Furthermore, H-16OH or H-16Ac evoked more c-Fos-positive neurons than L-16OH or L-16Ac, respectively, in the AOB of the mice of the same sex (Figure 7A).

**TABLE 1:** Effect of hexadecanol (16OH) and hexadecyl acetate (16Ac) on neuronal c-Fos immunoreaction in accessory olfactory bulb and brain nuclei of mice.

**Brain region**	**Male mice**					**Female mice**				
	**Control (*n* = 6)**	**L-16OH (*n* = 6)**	**H-16OH (*n* = 6)**	**L-16Ac (*n* = 6)**	**H-16Ac (*n* = 6)**	**Control (*n* = 6)**	**L-16OH (*n* = 6)**	**H-16OH (*n* = 6)**	**L-16Ac (*n* = 6)**	**H-16Ac (*n* = 6)**
AOB	100.4 ± 5.9	279.0 ± 14.3^c^	429.7 ± 17.3^c^	219.7 ± 5.0^c^	525.2 ± 804^c^	133.9 ± 7.7	345.0 ± 4.3^c^	745.8 ± 27.2^c^	175.4 ± 1.0	243.0 ± 9.7^c^
aAOB	51.4 ± 9.1	161.8 ± 23.3^b^	235.3 ± 24.5^a^	126.9 ± 7.3^b^	304.7 ± 12.9^c^	69.3 ± 10.5	202.5 ± 9.9^c^	409.5 ± 27.4^c^	101.1 ± 3.0^c^	137.9 ± 8.5^c^
pAOB	49.0 ± 5.95	117.2 ± 14.5	194.4 ± 21.5	92.7 ± 10.0	220.5 ± 16.0	64.5 ± 9.1	142.4 ± 8.4	336.3 ± 40.7	74.2 ± 2.2	105.1 ± 16.5
BNST	36.5 ± 6.3	129.1 ± 22.4	191.9 ± 13.6^b^	120.2 ± 10.5	306.6 ± 16.5^c^	38.8 ± 5.0	136.4 ± 6.7	494.8 ± 69.6^c^	101.2 ± 9.8	114.6 ± 15.5
MeA	57.2 ± 7.7	217.8 ± 61.0	287.6 ± 35.0^a^	297.7 ± 62.8^b^	595.5 ± 51.3^c^	76.5 ± 14.1	178.9 ± 19.9	718.2 ± 34.9^c^	186.3 ± 43.4	208.0 ± 36.8
PMCo	35.9 ± 6.5	125.6 ± 26.4	267.3 ± 36.0	173.5 ± 51.3	574.9 ± 79.1^c^	47.1 ± 10.3	124.5 ± 15.9	814.7 ± 94.5^c^	106.3 ± 7.2	166.4 ± 27.1
MPA	36.3 ± 5.4	126.5 ± 19.1	199.9 ± 11.2^b^	187.1 ± 25.4^a^	256.6 ± 12.6^c^	61.9 ± 9.1	126.7 ± 9.0	586.5 ± 48.3^c^	157.0 ± 19.0	171.0 ± 26.9^a^
VmH	54.7 ± 8.4	163.9 ± 25.0	278.0 ± 31.3^c^	234.9 ± 32.9^c^	331.3 ± 15.8^c^	47.6 ± 5.3	161.1 ± 22.1	629.6 ± 46.1^c^	167.1 ± 29.8	174.1 ± 16.0

*Data are expressed as the mean number of c-Fos+ neurons ± s.e.m. in the AOB and other brain regions of the male and female mice after exposure to 16OH and 16Ac at 10 nM (L) or 100 μM (H). ^*a*^*p* < 0.05, ^*b*^*p* < 0.01, ^*c*^*p* < 0.001. The comparison between each group and control (mineral oil) was assessed by one-way ANOVA LSD analysis while the comparison between aAOB and pAOB in the same group was analyzed by paired-sample *T*-test using PASW Statistics 18.0. The aAOB was compared with pAOB in the same group, and groups of the same sex were compared with control as well. AOB, accessory olfactory bulb; aAOB, anterior AOB; pAOB, posterior AOB; BNST, bed nucleus of the stria terminalis; MPA, medial preoptic area; MeA, medial amygdaloid nucleus; VmH, ventromedial hypothalamic nucleus; PMCo, posteromedial cortical amygdaloid nucleus.*

When the data from the aAOB and pAOB were processed separately, we found that 16OH and 16Ac induced stronger c-Fos expression at both low and high concentrations than control, except that in the female pAOB, L-16Ac failed to do so. Further, H-16OH and H-16Ac were more effective in eliciting c-Fos responses than their low counterparts in both male and female, aAOB or pAOB (Figures 7B,C).

As for sexual differences, the female AOB had more c-Fos+ cells than the male AOB in response to both L- and H-16OH whereas the male AOB had more c-Fos+ cells than the female AOB in response to both L- and H-16Ac. The pattern still held even when the data from the two AOB halves were analyzed separately: the female aAOB and pAOB responded significantly stronger than male aAOB and pAOB to both L- and H-16OH whereas in response to L- and H-16Ac, male aAOB and pAOB had more c-Fos+ neurons than their female counterparts (Figures 7D–F).

Comparative analysis of the efficacy of the two pheromones indicated that on the female AOB, L- or H-16OH was more effective than L- or H-16Ac whereas on the male AOB, L- or H-16Ac was more effective than L- or H-16OH. And the pattern was still true if the c-Fos immunostaining data were analyzed for aAOB and pAOB separately except the male pAOB (Figures 7G–I).

Following the initial processing in the AOB, pheromonal signals are known to be sent to the next orders of the neurons in several brain regions, including the BNST, MeA, PMCo, and then from both BNST and amygdaloid nuclei further downstream to the MPA and VmH (Supplementary Figure 1; Dulac and Wagner, 2006). To determine how the 16OH and 16Ac signals are processed in the higher brain regions, we carried out c-Fos immunohistochemistry on the brain sections containing the aforementioned nuclei to identify the neurons constituting the neural circuitry for these two pheromones.

### 16OH and 16Ac Induce c-Fos Immunoreactivity in the BNST

**FIGURE 8:**
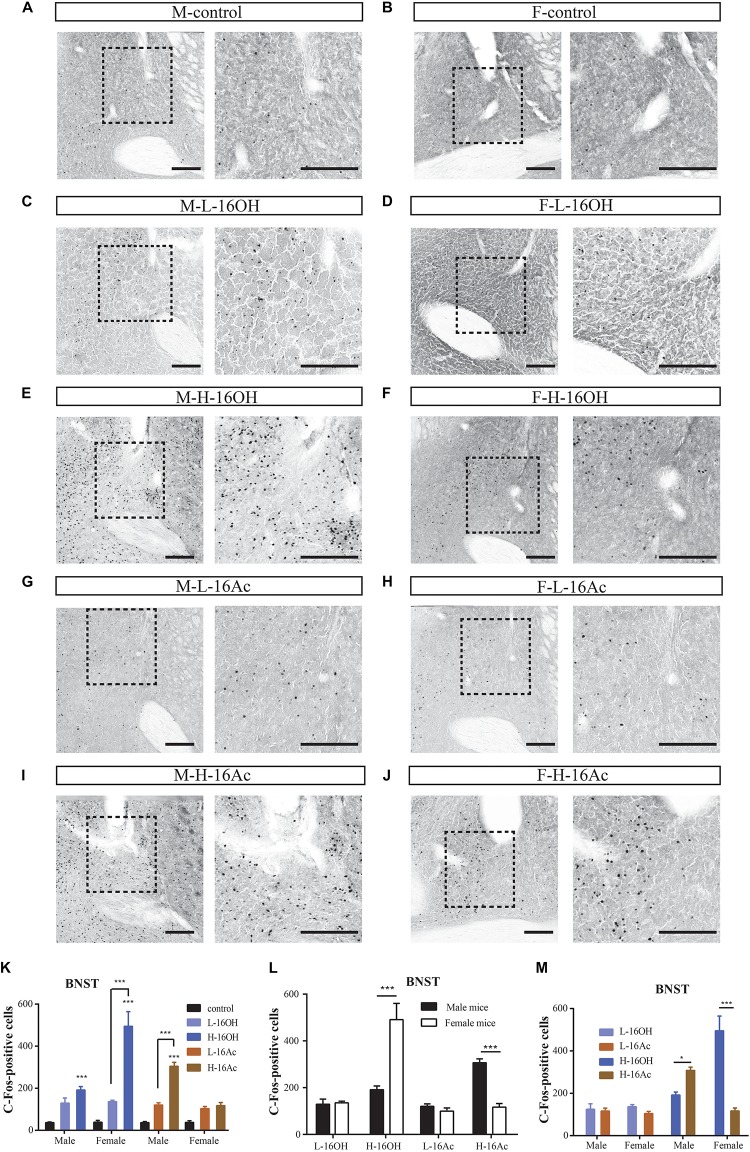
Representative images and quantification of c-Fos+ neurons in the bed nucleus of the stria terminalis (BNST) induced by 16OH and 16Ac. **(A–J)** Representative images and their insets of c-Fos+ cells in the BNST of male or female mice after exposure to 0.01% DCM-containing mineral oil (control), L-16OH, H-16OH, L-16Ac, and H-16Ac, respectively. Scale bar, 200 μm. **(K)** All treatments of H-16OH and H-16AC except H-16Ac on the female BNST evoked significantly stronger c-Fos immunoreactivity in the male and female BNST than the control (M-L-16OH vs. M-control, *t* = 2.598, *p* = 0.123; M-H-16OH vs. M-control, *t* = 4.358, *p* = 0.000; M-L-16Ac vs. M-control, *t* = 2.348, *p* = 0.228; M-H-16Ac vs. M-control, *t* = 7.577, *p* = 0.000; F-L-16OH vs. F-control, *t* = 2.737, *p* = 0.086; F-H-16OH vs. F-control, *t* = 12.793, *p* = 0.000; F-L-16Ac vs. F-control, *t* = 1.752, *p* = 0.860; F-H-16Ac vs. F-control, *t* = 2.172, *p* = 0.384). And H-16OH evoked significantly stronger c-Fos immunoreactivity than L-16OH in the female but not male mice whereas H-16Ac did so than L-16Ac in the opposite sex (F-H-16OH vs. F-L-16OH, *t* = 10.056, *p* = 0.000; M-H-16OH vs. M-L-16OH, *t* = 1.760, *p* = 0.845; M-H-16Ac vs. M-L-16Ac, *t* = 5.229, *p* = 0.000; F-H-16Ac vs. F-L-16Ac, *t* = 0.375, *p* = 1.000). **(L)** Comparison between sexes indicates that the female BNST responded more strongly to H-16OH than the male BNST whereas the male BNST did so to H-16Ac than the female BNST (F-H-16OH vs. M-H-16OH, *t* = 8.499, *p* = 0.000; M-H-16Ac vs. F-H-16Ac, *t* = 5.387, *p* = 0.000). **(M)** Comparison between the two pheromones indicates that H-16OH was more effective than H-16Ac on the female BNST whereas H-16Ac was more so than H-16OH on the male BNST (F-H-16OH vs. F-H-16Ac, *t* = 10.667, *p* = 0.000; M-H-16Ac vs. M-H-16OH, *t* = 3.219, *p* = 0.023). *N* = 6 for each group. All values are expressed as mean ± s.e.m. ^∗^*p* < 0.05, ^∗∗∗^*p* < 0.001.

After exposure to low and high 16OH and 16Ac, the male and female mice were examined for the c-Fos-immunoreactivity in the BNST (Figure 8). The results showed that in comparison with the 0.01% DCM-containing mineral oil-treated control samples, either L-16OH or L-16Ac could evoke significantly more c-Fos signals from male or female BNST where H-16OH and H-16Ac were able to do so on both male and female BNST except H-16Ac on the female BNST. And H-16OH and H-16Ac induced significantly more c-Fos+ cells than their counterparts of the low concentration on both male and female BNST except H-16Ac on the female BNST (Figure 8K and Table 1).

Comparison between sexes indicates that the female BNST responded more strongly to H-16OH than the male BNST whereas the male BNST did to H-16Ac than the female (Figure 8L). Comparison between the two pheromones indicates that H-16OH was more effective than H-16Ac on the female BNST whereas H-16Ac was more so than H-16OH on the male BNST (Figure 8M).

### 16OH and 16Ac Activate Neurons in the Medial Amygdaloid Nucleus

**FIGURE 9:**
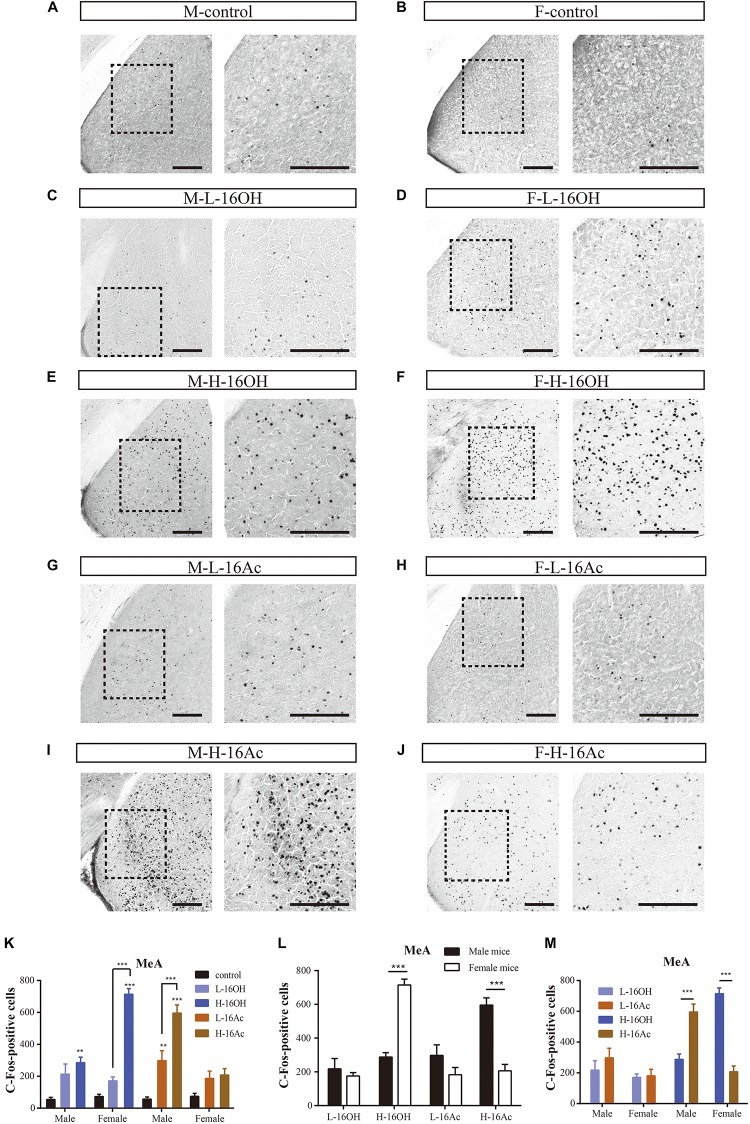
Representative images and quantification of c-Fos+ neurons in the anterior subdivision of the medial amygdaloid nucleus (MeA). **(A–J)** Representative images and their insets of c-Fos+ cells in the male and female MeA after exposure to 0.01% DCM-containing mineral oil (control), L- and H-16OH, and L- and H-16Ac. Scale bar, 200 μm. **(K–M)** Comparative analyses of the numbers of the c-Fos+ cells in the MeA between different concentrations of 16OH and 16Ac, between sexes or between the two pheromones, respectively. All four stimuli except L-16OH were able to elicit stronger c-Fos immunoreactivity than control in the male MeA whereas in the female MeA, only H-16-Ac was able to do so. H-16OH was significantly more effective than L-16OH on the female MeA whereas H-16Ac was more so than L-16Ac on the male MeA (M-L-16OH vs. M-control, *t* = 2.278, *p* = 0.075; M-H-16OH vs. M-control, *t* = 3.998, *p* = 0.002; M-L-16Ac vs. M-control, *t* = 4.172, *p* = 0.001; M-H-16Ac vs. M-control, *t* = 9.339, *p* = 0.000; F-L-16OH vs. F-control, *t* = 1.776, *p* = 0.817; F-H-16OH vs. F-control, *t* = 11.134, *p* = 0.000; F-L-16Ac vs. F-control, *t* = 1.905, *p* = 0.626; F-H-16Ac vs. F-control, *t* = 2.281, *p* = 0.268; F-H-16OH vs. F-L-16OH, *t* = 9.357, *p* = 0.000; M-H-16OH vs. M-L-16OH, *t* = 1.210, *p* = 1.000; M-H-16Ac vs. M-L-16Ac, *t* = 5.166, *p* = 0.000; F-H-16Ac vs. F-L-16Ac, *t* = 0.376, *p* = 1.000). Female MeA was more responsive to H-16OH whereas male MeA was more so to H-16Ac; and H-16OH was more effective than H-16Ac on the female MeA whereas on the male MeA, the opposite was true (F-H-16OH vs. M-H-16OH, *t* = 7.471, *p* = 0.000; M-H-16Ac vs. F-H-16Ac, *t* = 6.722, *p* = 0.000; F-L-16OH vs. M-L-16OH, *t* = 0.676, *p* = 0.502; M-L-16Ac vs. F-L-16Ac, *t* = 1.933, *p* = 0.059; F-H-16OH vs. F-H-16Ac, *t* = 8.852, *p* = 0.000; M-H-16Ac vs. M-H-16OH, *t* = 5.341, *p* = 0.000; F-L-16OH vs. F-L-16Ac, *t* = 0.128, *p* = 1.000; M-L-16Ac vs. M-L-16OH, *t* = 1.385, *p* = 1.000). *N* = 6 for each group. All values are expressed as mean ± s.e.m. ^∗∗^*p* < 0.01, ^∗∗∗^*p* < 0.001.

A major portion of the AOB output is known to be relayed to the MeA. To determine how the neurons in the nucleus are activated by 16OH and 16Ac, c-Fos immunoreactivity was also examined on the anterior subdivision of this nucleus (Figure 9). The results showed that the c-Fos immunoreactivity pattern mirrored, to some extent, that found in the BNST, e.g., in the male MeA, both L- and H-16Ac and H-16OH were able to elicit more c-Fos immunoreactivity than control, but only H-16Ac was more effective than L-16Ac in the male, whereas, in the female MeA, only H-16OH was able to evoke significantly more c-Fos immunoreactivity than control. However, both L-16OH and L- and H-16Ac failed to augment c-Fos response in the female MeA. Comparison between sexes or between the two pheromones lead to the similar results as found in the BNST, that is, the female MeA more robustly responded to H-16OH whereas the male MeA did to H-16Ac; and H-16OH was more effective than H-16Ac on the female MeA whereas the opposite was true on the male MeA (Figure 9 and Table 1).

### 16OH and 16Ac Activate Neurons in the Posteromedial Cortical Amygdaloid Nucleus

**FIGURE 10:**
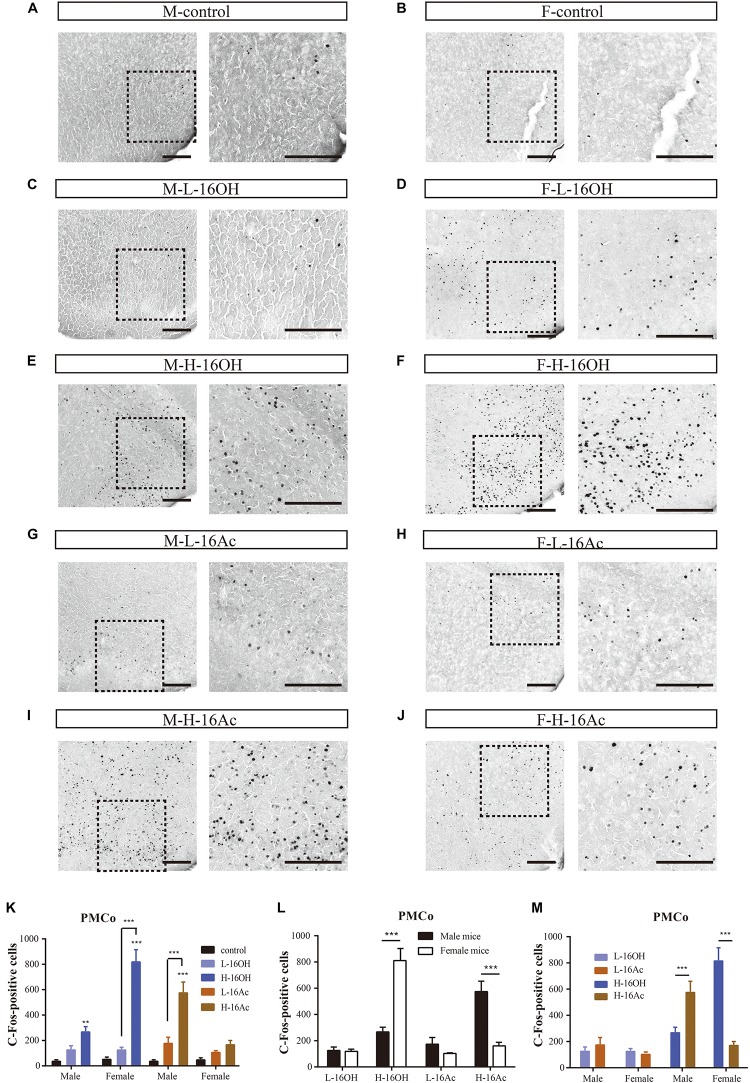
Representative images and quantification of the c-Fos+ neurons in the posteromedial cortical amygdaloid nucleus (PMCo). **(A–J)** Images and their insets of c-Fos-positive cells in the PMCo of male or female mice after exposure to 0.01% DCM-containing mineral oil (control), L- and H-16OH, and L- and H-16Ac, respectively. Scale bar, 200 μm. **(K)** H-16OH but not L-16OH was able to induce more c-Fos signals from both male and female PMCo; and H-16Ac produced significantly more c-Fos signals than control from the male but not female PMCo whereas L-16Ac were ineffective on either male or female PMCo (M-L-16OH vs. M-control, *t* = 1.384, *p* = 1.000; M-H-16OH vs. M-control, *t* = 3.570, *p* = 0.008; M-L-16Ac vs. M-control, *t* = 2.122, *p* = 0.388; M-H-16Ac vs. M-control, *t* = 8.317, *p* = 0.000; F-L-16OH vs. F-control, *t* = 1.193, *p* = 1.000; F-H-16OH vs. F-control, *t* = 11.845, *p* = 0.000; F-L-16Ac vs. F-control, *t* = 0.913, *p* = 1.000; F-H-16Ac vs. F-control, *t* = 1.841, *p* = 0.716; M-H-16OH vs. M-L-16OH, *t* = 2.187, *p* = 0.335; F-H-16OH vs. F-L-16OH, *t* = 10.651, *p* = 0.000; M-H-16Ac vs. M-L-16Ac, *t* = 6.194, *p* = 0.000; F-H-16Ac vs. F-L-16Ac, *t* = 0.927, *p* = 1.000). **(L,M)** The female PMCo was more sensitive than the male to H-16OH whereas the male PMCo was more so to H-16Ac than the female; and H-16OH was more effective than H-16Ac on the female MeA whereas on the male MeA, the opposite was true (F-H-16OH vs. M-H-16OH, *t* = 8.447, *p* = 0.000; M-H-16Ac vs. F-H-16Ac, *t* = 6.303, *p* = 0.000; F-L-16OH vs. M-L-16OH, *t* = 0.0175, *p* = 0.986; M-L-16Ac vs. F-L-16Ac, *t* = 1.037, *p* = 0.305; F-H-16OH vs. F-H-16Ac, *t* = 10.004, *p* = 0.000; M-H-16Ac vs. M-H-16OH, *t* = 4.746, *p* = 0.000; F-L-16OH vs. F-L-16Ac, *t* = 0.280, *p* = 1.000; M-L-16Ac vs. F-L-16Ac, *t* = 0.739, *p* = 1.000). *N* = 6 for each group. All values are expressed as mean ± s.e.m. ^∗∗^*p* < 0.01, ^∗∗∗^*p* < 0.001.

The PMCo is another nucleus that directly receives signals from the AOB. Comparative analyses showed that H-16OH but not L-16OH was able to produce significantly more c-Fos immunoreactivity in both male and female PMCo than control whereas only H-16Ac produced significantly more c-Fos signals than control in the male but not female PMCo with L-16Ac ineffective on either sex. And H-16Ac was more effective than L-16Ac on the male PMCo whereas H-16OH was more so than L-16OH on the female PMCo (Figure 10 and Table 1). Comparison between sexes or between the two pheromones lead to the same results as with the BNST or MeA, that is, the female PMCo was more sensitive to H-16OH whereas the male PMCo was to H-16Ac; and H-16OH was more effective than H-16Ac on the female PMCo whereas the opposite was true on the male PMCo (Figure 10 and Table 1).

### 16OH and 16Ac Activate Neurons in the Medial Preoptic Area

**FIGURE 11:**
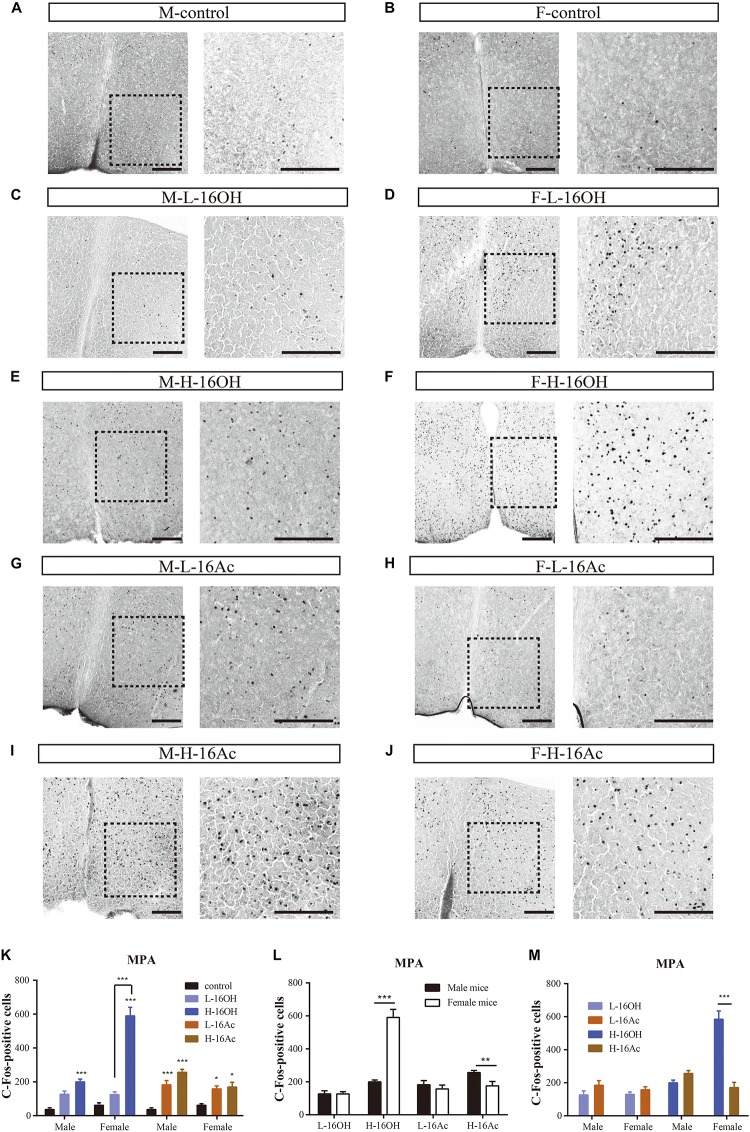
Representative images and quantification of c-Fos+ neurons in the medial preoptic area (MPA). **(A–J)** Images and their insets of c-Fos+ cells in MPA of male or female mice after exposure to 0.01% DCM-containing mineral oil (control), L- and H-16OH, and L- and H-16Ac, respectively. Scale bar, 200 μm. **(K–M)** Comparative analysis of the c-Fos data between different concentrations of the pheromones, between sexes, and between the two pheromones, respectively. All four stimuli except L-16OH induced significantly more c-Fos+ cells in the male and female MPA; and H-16OH evoked more than L-16OH on the female but not male MPA whereas H-16Ac did not elicit more c-Fos+ cells than L-16Ac in the MPA of either sex (M-L-16OH vs. M-control, *t* = 2.833, *p* = 0.058; M-H-16OH vs. M-control, *t* = 5.266, *p* = 0.000; M-L-16Ac vs. M-control, *t* = 4.658, *p* = 0.000; M-H-16Ac vs. M-control, *t* = 7.038, *p* = 0.000; F-L-16OH vs. F-control, *t* = 2.069, *p* = 0.438; F-H-16OH vs. F-control, *t* = 16.756, *p* = 0.000; F-L-16Ac vs. F-control, *t* = 3.036, *p* = 0.038; F-H-16Ac vs. F-control, *t* = 3.458, *p* = 0.010; M-H-16OH vs. M-L-16OH, *t* = 2.343, *p* = 0.231; F-H-16OH vs. F-L-16OH, *t* = 14.687, *p* = 0.000; M-H-16Ac vs. M-L-16Ac, *t* = 2.381, *p* = 0.211; F-H-16Ac vs. F-L-16Ac, *t* = 0.448, *p* = 1.000). H-16OH was more effective on the female MPA than on the male MPA whereas H-16Ac was more so on the male MPA than on the female one (F-H-16OH vs. M-H-16OH, *t* = 12.348, *p* = 0.000; M-H-16Ac vs. F-H-16Ac, *t* = 2.735, *p* = 0.009; F-L-16OH vs. M-L-16OH, *t* = 0.00426, *p* = 0.997; M-L-16Ac vs. F-L-16Ac, *t* = 0.803, *p* = 0.426). And H-16OH seemed to be more effective than H-16Ac on the female MPA (M-L-16OH vs. M-L-16Ac, *t* = 1.775, *p* = 0.820; M-H-16OH vs. M-H-16Ac, *t* = 1.812, *p* = 0.760; F-L-16OH vs. F-L-16Ac, *t* = 0.968, *p* = 1.000; F-H-16OH vs. F-H-16Ac, *t* = 13.271, *p* = 0.000). *N* = 6 for each group. All values are expressed as mean ± s.e.m. ^∗^*p* < 0.05, ^∗∗^*p* < 0.01, ^∗∗∗^*p* < 0.001.

The MPA is known to receive signals from both the BNST and MeA. To determine whether 16OH and 16Ac also activate the neurons in this nucleus, we performed c-Fos immunostaining and analyzed the data (Figure 11). The results showed that both male and female mice generated significantly more c-Fos immunoreactivity in the nuclei following the exposure to L-16OH, H-16OH, or H-16Ac than the control (Figure 11 and Table 1). Furthermore, H-16OH evoked more immunoreactivity than L-16OH in the female but not male MPA while H-16Ac was not significantly more effective than L-16Ac on either male or female MPA. Comparison between sexes indicated that the female MPA was more sensitive to H-16OH than its male counterpart while the opposite was true in response to H-16Ac. Comparison between the two pheromones indicated that only H-16OH was more effective than H-16Ac on the female MPA and no any other significant differences were found (Figure 11).

### 16OH and 16Ac Activate Neurons in the Ventromedial Hypothalamic Nuclei

**FIGURE 12:**
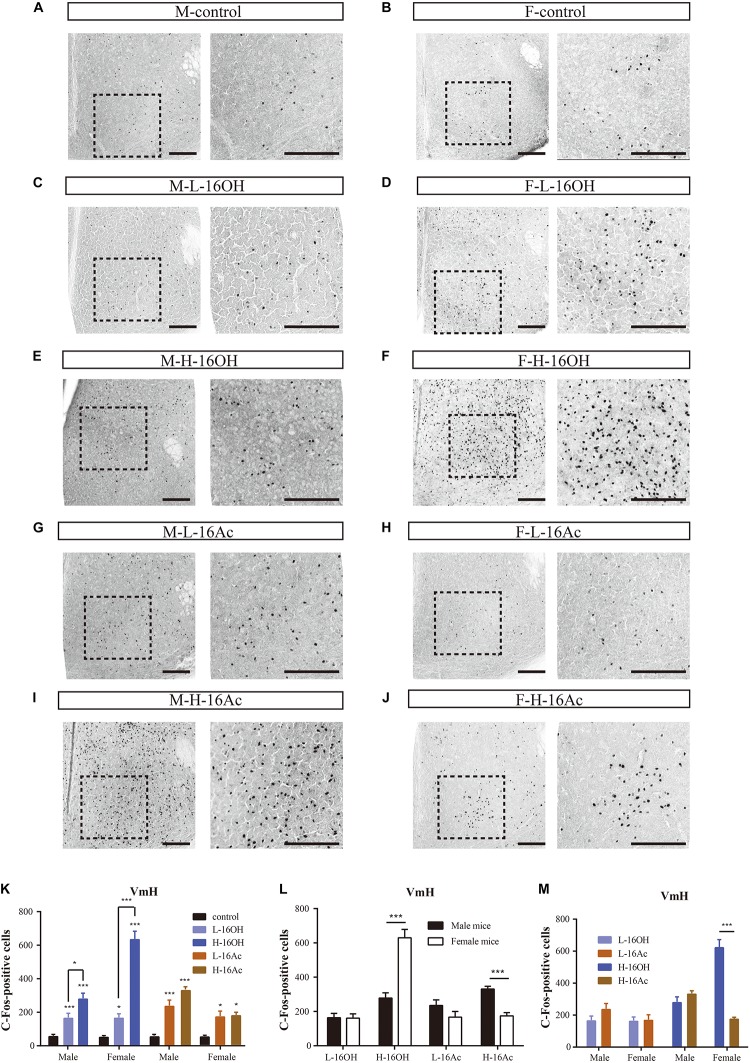
Representative images and quantification of the c-Fos+ neurons in the ventromedial hypothalamic nucleus (VmH). **(A–J)** Images and their insets of c-Fos+ cells in the VmH of male or female mice after exposure to 0.01% DCM-containing mineral oil (control), L- and H-16OH, and L- and H-16Ac. Scale bar, 200 μm. **(K)** L-16OH, H-16OH, L-16Ac, or H-16Ac generated more c-Fos signals than the control on both male and female VmH; H-16OH was more effective than L-16OH on both male and female VmH whereas H-16Ac did not elicit more c-Fos signals than L-16Ac on either male or female VmH (M-L-16OH vs. M-control, *t* = 2.965, *p* = 0.000; M-H-16OH vs. M-control, *t* = 6.602, *p* = 0.000; M-L-16Ac vs. M-control, *t* = 4.892, *p* = 0.000; M-H-16Ac vs. M-control, *t* = 7.509, *p* = 0.000; F-L-16OH vs. F-control, *t* = 3.082, *p* = 0.032; F-H-16OH vs. F-control, *t* = 15.797, *p* = 0.000; F-L-16Ac vs. F-control, *t* = 3.243, *p* = 0.021; F-H-16Ac vs. F-control, *t* = 3.435, *p* = 0.012; M-H-16OH vs. M-L-16OH; *t* = 3.097, *p* = 0.032; F-H-16OH vs. F-L-16OH, *t* = 12.715, *p* = 0.000; M-H-16Ac vs. M-L-16Ac, *t* = 2.617, *p* = 0.117; F-H-16Ac vs. F-L-16Ac, *t* = 0.192, *p* = 1.000). **(L,M)** The female VmH was more responsive than the male VmH to H-16OH whereas the opposite was true to H-16Ac. H-16OH was more effective than H-16Ac on the female VmH (F-H-16OH vs. M-H-16OH, *t* = 9.543, *p* = 0.000; M-H-16Ac vs. F-H-16Ac, *t* = 4.266, *p* = 0.000; F-L-16OH vs. M-L-16OH, *t* = 0.0751, *p* = 0.940; F-L-16Ac vs. M-L-16Ac, *t* = 1.841, *p* = 0.072; F-H-16OH vs. F-H-16Ac, *t* = 12.362, *p* = 0.000; M-H-16OH vs. M-H-16Ac, *t* = 1.447, *p* = 1.000; F-L-16OH vs. F-L-16Ac, *t* = 0.161, *p* = 1.000; M-L-16OH vs. M-L-16Ac, *t* = 1.927, *p* = 0.596). *N* = 6 for each group. All values are expressed as mean ± s.e.m. ^∗^*p* < 0.05, ^∗∗∗^*p* < 0.001.

The signals from the MPA are known to be relayed to the VmH. c-Fos immunoreactivity analyses showed that the response patterns were nearly identical to those found in the MPA, that is, all stimulations, L- or H-16OH, L- or H-16Ac generated more c-Fos signals than control on both male and female VmH (Figure 12 and Table 1). But the concentration-dependent increases in c-Fos signals were found for 16OH on both male and female VmH only. The female VmH was more sensitive than the male VmH to H-16OH whereas the opposite was true to H-16Ac. H-16OH was more effective than H-16Ac on the female VmH (Figure 12).

## Discussion

The mammalian olfactory system is comprised of two subsystems: the MOS and the AOS. While the former is mainly responsible for detecting volatile odorants, the latter is largely for detecting less or non-volatile pheromones, although some cross-detections between these two have been reported as well (Holy, 2018; Mohrhardt et al., 2018). Unlike a relatively large number of odorants and their corresponding olfactory receptors, very few pheromones or their receptors have been structurally identified or deorphanized. Hexadecanol (16OH) and hexadecyl acetate (16Ac) are two semi-volatile and rare chemically pure pheromones that exert differential effects on male and female mice (Zhang et al., 2008). In-depth studies on these two pheromones can facilitate our understanding of the AOS mechanisms.

Calcium imaging of the VNO slices indicated that a small percentage (0.6–1.9%) of male and female VSNs responded to 16OH only, 16Ac only, or both 16OH and 16Ac (Figures 1–4), which is very similar to the percentages of VSNs activated by several other known pheromones, including the small molecules: 2,5-dimethypyrazine, SBT, 2,3-dehydro-exo-brevicomin, α- and β-farnesenes, 2-heptanone, and HMH, and the large molecules such as peptides ESPs and major urine proteins (Leinders-Zufall et al., 2000; Kimoto et al., 2007; Ferrero et al., 2013; Dey et al., 2015). Notably, only one vomeronasal receptor has been identified for each of these pheromones: Vmn1r49 for 2-heptanone, Vmn2r116 for ESP1, and V2Rp4 for ESP22 (Boschat et al., 2002; Haga et al., 2010; Osakada et al., 2018). Thus, given the similar percentages of the activated VSNs, we conclude that it is likely only one vomeronasal receptor each for detecting 16OH, 16Ac, or both.

Further, our data also showed that the VSNs responsive to 16OH, 16Ac, or both were preferentially located in the apical VSE, and that the numbers of responsive neurons did not increase in most cases even when the pheromone concentrations were raised by 10,000-fold, from 10 nM to 100 μM, with one exception of the female VSNs that significantly increased by twofold upon stimulation of 100 μM 16OH, i.e., H-16OH (Figures 2–5). These results indicate that probably there is only one V1r in the male and female VSE sensing 16OH, 16Ac, or both, respectively, whereas the female VSNs may express an additional V1r for detecting the high concentration of 16OH. On the other hand, however, the basal VSNs also responded, to a lesser extent, to these chemicals. Thus, it is also possible that there is another V2r responsive to each of 16OH and 16Ac or both, respectively.

The pheromonal signals detected by VSNs are then projected to the AOB. Our c-Fos immunostaining data indicate that significantly more c-Fos+ neurons were found in the aAOB than in the pAOB (Figure 6), which is in congruency with the calcium imaging data showing the apical location of most responsive VSNs (Figure 5). However, the male pAOB responded to all four stimuli: L- and H-16OH, L- and H-16Ac whereas the female pAOB did to three of the four: L- and H-16OH, and H-16Ac, but not to L-16Ac (Figure 7), which is also consistent with the VNO results showing calcium responses also from the basal VSNs to 16OH and 16Ac, or both, supporting the idea of V2r responsive to these two pheromones.

The numbers of male and female c-Fos+ neurons in the AOB, aAOB, or pAOB were 16OH and 16Ac dose-dependent (Figure 7), which is different from the observation on the VSE, of which only the number of the female 16OH-responding VSNs was 16OH concentration-dependent. This discrepancy may be explained by the fact that VSN firing rates vary from 2.5 to 80 Hz (Stowers et al., 2002; Nodari et al., 2008; Kim et al., 2012), and that individual VSNs innervate multiple AOB glomeruli; conversely, some individual glomeruli receive projections from multiple VSNs expressing different vomeronasal receptors (Belluscio et al., 1999; Rodriguez et al., 1999; Wagner et al., 2006), although a mitral cell tends to receive inputs from different glomeruli that are innervated by sensory neurons expressing the same type of V1r or V2r receptor (Del Punta et al., 2002). Thus, high concentrations of 16OH and 16Ac may trigger more spikes from VSNs, increasing firing rate, which cannot be detected by the VSN calcium imaging, but activate more glomeruli, generating more c-Fos+ AOB neurons. Further studies, however, are needed to validate this notion.

Mitral cells in the AOB send their axons to other AOS circuit hubs in the brain. Interestingly, the BNST receives inputs only from the aAOB, not from pAOB (Martinez-Marcos and Halpern, 1999; Mohedano-Moriano et al., 2007). The c-Fos immunoreactivity pattern in the BNST was closely similar to that in the AOB: the male BNST exhibited significantly more c-Fos+ neurons in response to H-16OH and H-16Ac whereas the female BNST showed significant responses to H-16OH (Figure 8). These results indicate that the information is relayed largely intact from the AOB to BNST in response to the high concentration of 16OH or 16Ac.

Mitral cells in the AOB can also send axons to the MeA and PMCo, followed by the projection of BNST and MeA neurons to the MPA, and finally to the VmH. The overall profiles of c-Fos immunoreactivity in these nuclei were similar to that found in the BNST, and can be summarized as following: (1) dose-dependency: stronger c-Fos responses were induced by higher concentrations of 16OH and 16Ac than lower ones, respectively; (2) sexual dimorphism: the female nuclei responded more strongly to 16OH whereas male nuclei did so to 16Ac; and (3) the relative effectiveness between the two pheromones: significant differences in evoking c-Fos responses in the nuclei were found between the two pheromones only at their high concentrations but not at the low concentrations. There are, however, some subtle variations. For example, the female MeA exhibited insignificant increases in the number of c-Fos+ neurons in response to not only L- or H-16Ac but also L-16OH, as in the BNST and PMCo (Figures 9, 10), whereas the male BNST and PMCo nuclei did not respond to L-16OH or L-16Ac. In the MPA and VmH, males responded equally to H-16OH and H-16Ac.

Neurons in the nuclei examined so far in this study are involved in various pheromone-induced activities. For example, activation of some BNST neurons can suppress female sexual behavior (Osakada et al., 2018), and even the PMCo, which does not have much direct connections to the hypothalamic nuclei, process and relay most information from the AOB to the MeA and posterolateral amygdaloid cortex (Gutierrez-Castellanos et al., 2014). The MeA, which receives excitatory signals from the AOB and the PMCo, sends axons to different amygdala and hypothalamic areas, directing sex-specific behavioral responses as well as endocrine and autonomic responses (Brennan and Zufall, 2006; Ishii et al., 2017). The MPA is known to be critical to control inter-male and pup-directed aggression, sexual behaviors, and parental care (Wu et al., 2014; Wei et al., 2018). Finally, some neurons in the VmH are involved in initiating aggression, parental caring, and mating as well (Lin et al., 2011; Kohl et al., 2018). However, the only documented behavioral responses induced by 16OH and 16Ac include attraction and aversion, with no evident aggression, mating, or parental caring observed yet (Zhang et al., 2008). Our c-Fos data indicate that 16OH- and 16Ac-activated neurons in the brain regions can be parts of the AOS circuit hubs, contributing to the modulation or fine-tuning of the behavioral outputs determined by these ensembles of neurons (Li et al., 2017; Li and Dulac, 2018). Further investigations are needed to unravel the exact roles played by the 16OH- and 16Ac-activated neurons in the population coding of the pheromonal signals.

Like many other pheromones, 16OH and 16Ac also evoke sexually dimorphic responses. In the VNO, similar numbers of male VSNs responded to both 16OH and 16Ac; in contrast, many more female VSNs did to 16OH than to 16Ac (Figures 1–3); and upon stimulation of H-16OH, far more VSNs were activated in females than in males (Figures 4, 5). In the AOB and most other brain regions, it seems largely consistent that males responded more strongly to 16Ac, females more strongly to 16OH, especially at the high concentration, except in the male MPA and VmH (Figures 6–12). It is known that AOS brain regions between sexes differ in neuron numbers and sex hormone receptor expression (Li and Dulac, 2018). The AOS can be modulated by the inputs from the MOS, by physiological states and experiences. For example, the enzyme aromatase can convert testosterone into estrogen, which acts on the neurons expressing the estrogen receptors, modulating their activity, while mating experience makes a subset of male MeA neurons more responsive (Dey et al., 2015; Xu et al., 2016; Lischinsky et al., 2017). Additional studies are needed to reveal the behavioral and physiological consequences resulted from or modulated by 16OH and 16Ac sexual dimorphic effects.

## Conclusion

In conclusion, our study demonstrates the activation of VSNs by 16OH and 16Ac, and the activation of neurons in the circuit hubs of the AOS in a sexually dimorphic manner. Our data indicate that there is one V1r each for 16OH, 16Ac, or both, and possibly another V1r for the concentrated 16OH. And activation of the neurons in the brain regions strongly suggests that 16OH and 16Ac can modulate the AOS outputs such as aggression, mating, and parental caring. Further studies on these two abundantly produced and chemically identified natural pheromones can facilitate our understanding of this ancient and vital system in many mammals.

## Data Availability Statement

All datasets generated for this study are included in the manuscript/Supplementary Files.

## Ethics Statement

The animal study was reviewed and approved by the Institutional Animal Care and Use Committees of both the Zhejiang University and the Institute of Zoology, Chinese Academy of Sciences.

## Author Contributions

QL, J-XZ, and LH designed the experiments, analyzed and interpreted the data, and wrote the manuscript. QL, XG, PW, YZ, and YW performed the experiments.

## Conflict of Interest

The authors declare that the research was conducted in the absence of any commercial or financial relationships that could be construed as a potential conflict of interest.

## References

[B1] Akiyoshi S. Ishii T. Bai Z. Mombaerts P. (2018). Subpopulations of vomeronasal sensory neurons with coordinated coexpression of type 2 vomeronasal receptor genes are differentially dependent on Vmn2r1. *Eur. J. Neurosci.* 47 887–900. 10.1111/ejn.13875 29465786 PMC5947554

[B2] Belluscio L. Koentges G. Axel R. Dulac C. (1999). A map of pheromone receptor activation in the mammalian brain. *Cell* 97 209–220. 10.1016/s0092-8674(00)80731-x 10219242

[B3] Boschat C. Pelofi C. Randin O. Roppolo D. Luscher C. Broillet M. C. (2002). Pheromone detection mediated by a V1r vomeronasal receptor. *Nat. Neurosci.* 5 1261–1262. 10.1038/nn978 12436115

[B4] Brechbühl J. Luyet G. Moine F. Rodriguez I. Broillet M.-C. (2011). Imaging pheromone sensing in a mouse vomeronasal acute tissue slice preparation. *J. Vis. Exp.* 58:e3311. 10.3791/3311 22157638 PMC3369656

[B5] Brennan P. A. Zufall F. (2006). Pheromonal communication in vertebrates. *Nature* 444 308–315. 10.1038/nature05404 17108955

[B6] Del Punta K. Puche A. Adams N. C. Rodriguez I. Mombaerts P. (2002). A divergent pattern of sensory axonal projections is rendered convergent by second-order neurons in the accessory olfactory bulb. *Neuron* 35 1057–1066. 10.1016/s0896-6273(02)00904-2 12354396

[B7] Dey S. Chamero P. Pru J. K. Chien M. S. Ibarra-Soria X. Spencer K. R. (2015). Cyclic regulation of sensory perception by a female hormone alters behavior. *Cell* 161 1334–1344. 10.1016/j.cell.2015.04.052 26046438 PMC4501503

[B8] Doyle W. I. Dinser J. A. Cansler H. L. Zhang X. Dinh D. D. Browder N. S. (2016). Faecal bile acids are natural ligands of the mouse accessory olfactory system. *Nat. Commun.* 7:11936. 10.1038/ncomms11936 27324439 PMC4919516

[B9] Dulac C. Torello A. T. (2003). Molecular detection of pheromone signals in mammals: from genes to behaviour. *Nat. Rev. Neurosci.* 4 551–562. 10.1038/nrn1140 12838330

[B10] Dulac C. Wagner S. (2006). Genetic analysis of brain circuits underlying pheromone signaling. *Ann. Rev. Genet.* 40 449–467. 10.1146/annurev.genet.39.073003.093937 16953793

[B11] Fabianová K. Martončíková M. Fabian D. Blaško J. Račeková E. (2014). Diverse effect of different odor stimuli on behavior and Fos protein production in the olfactory system neurogenic region of adult rats. *Behav. Brain Res.* 265(Suppl. C), 38–48. 10.1016/j.bbr.2014.01.023 24485916

[B12] Ferrero D. M. Moeller L. M. Osakada T. Horio N. Li Q. Roy D. S. (2013). A juvenile mouse pheromone inhibits sexual behaviour through the vomeronasal system. *Nature* 502 368–371. 10.1038/nature12579 24089208 PMC3800207

[B13] Gutierrez-Castellanos N. Pardo-Bellver C. Martinez-Garcia F. Lanuza E. (2014). The vomeronasal cortex - afferent and efferent projections of the posteromedial cortical nucleus of the amygdala in mice. *Eur. J. Neurosci.* 39 141–158. 10.1111/ejn.12393 24188795

[B14] Haga S. Hattori T. Sato T. Sato K. Matsuda S. Kobayakawa R. (2010). The male mouse pheromone ESP1 enhances female sexual receptive behaviour through a specific vomeronasal receptor. *Nature* 466 118–122. 10.1038/nature09142 20596023

[B15] Haga-Yamanaka S. Ma L. He J. Qiu Q. Lavis L. D. Looger L. L. (2014). Integrated action of pheromone signals in promoting courtship behavior in male mice. *eLife* 3:e03025. 10.7554/eLife.03025 25073926 PMC4107909

[B16] Halem H. A. Cherry J. A. Baum M. J. (1999). Vomeronasal neuroepithelium and forebrain Fos responses to male pheromones in male and female mice. *J. Neurobiol.* 39 249–263. 10.1002/(SICI)1097-4695(199905)39:2<249::AID-NEU9>3.0.CO;2-R 10235679

[B17] Halpern M. Martınez-Marcos A. (2003). Structure and function of the vomeronasal system: an update. *Prog. Neurobiol.* 70 245–318. 10.1016/s0301-0082(03)00103-5 12951145

[B18] Holy T. E. (2018). The accessory olfactory system: innately specialized or microcosm of mammalian circuitry? *Annu. Rev. Neurosci.* 41 501–525. 10.1146/annurev-neuro-080317-061916 29727596

[B19] Honda N. Sakamoto H. Inamura K. Kashiwayanagi M. (2008). Changes in Fos expression in the accessory olfactory bulb of sexually experienced male rats after exposure to female urinary pheromones. *Eur. J. Neurosci.* 27 1980–1988. 10.1111/j.1460-9568.2008.06169.x 18412619

[B20] Hurst J. L. Payne C. E. Nevison C. M. Marie A. D. Humphries R. E. Robertson D. H. (2001). Individual recognition in mice mediated by major urinary proteins. *Nature* 414 631–634. 10.1038/414631a 11740558

[B21] Ishii K. K. Osakada T. Mori H. Miyasaka N. Yoshihara Y. Miyamichi K. (2017). A labeled-line neural circuit for pheromone-mediated sexual behaviors in mice. *Neuron* 95:123-137.e8. 10.1016/j.neuron.2017.05.038 28648498

[B22] Isogai Y. Si S. Pont-Lezica L. Tan T. Kapoor V. Murthy V. N. (2011). Molecular organization of vomeronasal chemoreception. *Nature* 478 241–245. 10.1038/nature10437 21937988 PMC3192931

[B23] Isogai Y. Wu Z. Love M. I. Ahn M. H. Bambah-Mukku D. Hua V. (2018). Multisensory logic of infant-directed aggression by males. *Cell* 175:1827-1841.e17. 10.1016/j.cell.2018.11.032 30550786 PMC6558521

[B24] Jiang Y. Gong N. N. Hu X. S. Ni M. J. Pasi R. Matsunami H. (2015). Molecular profiling of activated olfactory neurons identifies odorant receptors for odors in vivo. *Nat. Neurosci.* 18 1446–1454. 10.1038/nn.4104 26322927 PMC4583814

[B25] Karlson P. Luscher M. (1959). Pheromones’: a new term for a class of biologically active substances. *Nature* 183 55–56. 10.1038/183055a0 13622694

[B26] Kaur A. W. Ackels T. Kuo T. H. Cichy A. Dey S. Hays C. (2014). Murine pheromone proteins constitute a context-dependent combinatorial code governing multiple social behaviors. *Cell* 157 676–688. 10.1016/j.cell.2014.02.025 24766811 PMC4051225

[B27] Keverne E. B. (1999). The vomeronasal organ. *Science* 286 716–720. 10.1126/science.286.5440.716 10531049

[B28] Kim S. Ma L. Jensen K. L. Kim M. M. Bond C. T. Adelman J. P. (2012). Paradoxical contribution of SK3 and GIRK channels to the activation of mouse vomeronasal organ. *Nat. Neurosci.* 15 1236–1244. 10.1038/nn.3173 22842147 PMC3431453

[B29] Kimoto H. Haga S. Sato K. Touhara K. (2005). Sex-specific peptides from exocrine glands stimulate mouse vomeronasal sensory neurons. *Nature* 437 898–901. 10.1038/nature04033 16208374

[B30] Kimoto H. Sato K. Nodari F. Haga S. Holy T. E. Touhara K. (2007). Sex- and strain-specific expression and vomeronasal activity of mouse ESP family peptides. *Curr. Biol.* 17 1879–1884. 10.1016/j.cub.2007.09.042 17935991

[B31] Kohl J. Babayan B. M. Rubinstein N. D. Autry A. E. Marin-Rodriguez B. Kapoor V. (2018). Functional circuit architecture underlying parental behaviour. *Nature* 556 326–331. 10.1038/s41586-018-0027-0 29643503 PMC5908752

[B32] Leinders-Zufall T. Brennan P. Widmayer P. Maul-Pavicic A. Jäger M. Li X.-H. (2004). MHC class I peptides as chemosensory signals in the vomeronasal organ. *Science* 306 1033–1037. 10.1126/science.1102818 15528444

[B33] Leinders-Zufall T. Ishii T. Chamero P. Hendrix P. Oboti L. Schmid A. (2014). A family of nonclassical class I MHC genes contributes to ultrasensitive chemodetection by mouse vomeronasal sensory neurons. *J. Neurosci.* 34 5121–5133. 10.1523/JNEUROSCI.0186-14.2014 24719092 PMC4050176

[B34] Leinders-Zufall T. Lane A. P. Puche A. C. Ma W. Novotny M. V. Shipley M. T. (2000). Ultrasensitive pheromone detection by mammalian vomeronasal neurons. *Nature* 405 792–796. 10.1038/35015572 10866200

[B35] Li Y. Dulac C. (2018). Neural coding of sex-specific social information in the mouse brain. *Curr. Opin. Neurobiol.* 53 120–130. 10.1016/j.conb.2018.07.005 30059820

[B36] Li Y. Mathis A. Grewe B. F. Osterhout J. A. Ahanonu B. Schnitzer M. J. (2017). Neuronal representation of social information in the medial amygdala of awake behaving mice. *Cell* 171:1176-1190.e17. 10.1016/j.cell.2017.10.015 29107332 PMC5731476

[B37] Liberles S. D. (2014). Mammalian pheromones. *Ann. Rev. Physiol.* 76 151–175. 10.1146/annurev-physiol-021113-170334 23988175 PMC4310675

[B38] Lin D. Boyle M. P. Dollar P. Lee H. Lein E. S. Perona P. (2011). Functional identification of an aggression locus in the mouse hypothalamus. *Nature* 470 221–226. 10.1038/nature09736 21307935 PMC3075820

[B39] Lischinsky J. E. Sokolowski K. Li P. Esumi S. Kamal Y. Goodrich M. (2017). Embryonic transcription factor expression in mice predicts medial amygdala neuronal identity and sex-specific responses to innate behavioral cues. *eLife* 6:e21012. 10.7554/eLife.21012 28244870 PMC5384829

[B40] Ma L. Haga-Yamanaka S. Yu Q. E. Qiu Q. Kim S. Yu C. R. (2011). Imaging neuronal responses in slice preparations of vomeronasal organ expressing a genetically encoded calcium sensor. *J. Vis. Exp.* 6:3404. 10.3791/3404 22157702 PMC3369649

[B41] Martinez-Marcos A. Halpern M. (1999). Differential projections from the anterior and posterior divisions of the accessory olfactory bulb to the medial amygdala in the opossum, *Monodelphis domestica*. *Eur. J. Neurosci.* 11 3789–3799. 10.1046/j.1460-9568.1999.00797.x 10583468

[B42] McElfresh J. S. Hammond A. M. Millar J. G. (2001). Sex pheromone components of the buck moth *Hemileuca maia*. *J. Chem. Ecol.* 27 1409–1422. 11504036 10.1023/a:1010369326866

[B43] Mohedano-Moriano A. Pro-Sistiaga P. Ubeda-Banon I. Crespo C. Insausti R. Martinez-Marcos A. (2007). Segregated pathways to the vomeronasal amygdala: differential projections from the anterior and posterior divisions of the accessory olfactory bulb. *Eur. J. Neurosci.* 25 2065–2080. 10.1111/j.1460-9568.2007.05472.x 17419754

[B44] Mohrhardt J. Nagel M. Fleck D. Ben-Shaul Y. Spehr M. (2018). Signal detection and coding in the accessory olfactory system. *Chem. Senses* 43 667–695. 10.1093/chemse/bjy061 30256909 PMC6211456

[B45] Munger S. D. Leinders-Zufall T. Zufall F. (2009). Subsystem organization of the mammalian sense of smell. *Ann. Rev. Physiol.* 71 115–140. 10.1146/annurev.physiol.70.113006.100608 18808328

[B46] Nodari F. Hsu F. F. Fu X. Holekamp T. F. Kao L. F. Turk J. (2008). Sulfated steroids as natural ligands of mouse pheromone-sensing neurons. *J. Neurosci.* 28 6407–6418. 10.1523/JNEUROSCI.1425-08.2008 18562612 PMC2726112

[B47] Novotny M. Harvey S. Jemiolo B. (1990). Chemistry of male dominance in the house mouse, *Mus domesticus*. *Experientia* 46 109–113. 10.1007/bf01955433 2298278

[B48] Novotny M. V. (2003). Pheromones, binding proteins and receptor responses in rodents. *Biochem. Soc. Trans.* 31 117–122. 10.1042/bst0310117 12546667

[B49] Novotny M. V. Jemiolo B. Wiesler D. Ma W. Harvey S. Xu F. (1999). A unique urinary constituent, 6-hydroxy-6-methyl-3-heptanone, is a pheromone that accelerates puberty in female mice. *Chem. Biol.* 6 377–383. 10.1016/S1074-5521(99)80049-0 10375540

[B50] Osakada T. Ishii K. K. Mori H. Eguchi R. Ferrero D. M. Yoshihara Y. (2018). Sexual rejection via a vomeronasal receptor-triggered limbic circuit. *Nat. Commun.* 9:4463. 10.1038/s41467-018-07003-5 30367054 PMC6203846

[B51] Paxinos G. Franklin K. B. J. (2004). *The Mouse Brain in Stereotaxic Coordinates.* Amsterdam: Elsevier Academic Press.

[B52] Riviere S. Challet L. Fluegge D. Spehr M. Rodriguez I. (2009). Formyl peptide receptor-like proteins are a novel family of vomeronasal chemosensors. *Nature* 459 574–577. 10.1038/nature08029 19387439

[B53] Rodriguez I. Feinstein P. Mombaerts P. (1999). Variable patterns of axonal projections of sensory neurons in the mouse vomeronasal system. *Cell* 97 199–208. 10.1016/s0092-8674(00)80730-8 10219241

[B54] Stowers L. Holy T. E. Meister M. Dulac C. Koentges G. (2002). Loss of sex discrimination and male-male aggression in mice deficient for TRP2. *Science* 295 1493–1500. 10.1126/science.1069259 11823606

[B55] Sturm T. Leinders-Zufall T. Macek B. Walzer M. Jung S. Pommerl B. (2013). Mouse urinary peptides provide a molecular basis for genotype discrimination by nasal sensory neurons. *Nat. Commun.* 4:1616. 10.1038/ncomms2610 23511480

[B56] Szymanski L. A. Keller M. (2014). Activation of the olfactory system in response to male odors in female prepubertal mice. *Behav. Brain Res.* 271(Suppl. C), 30–38. 10.1016/j.bbr.2014.05.051 24886778

[B57] Touhara K. Vosshall L. B. (2009). Sensing odorants and pheromones with chemosensory receptors. *Ann. Rev. Physiol.* 71 307–332. 10.1146/annurev.physiol.010908.163209 19575682

[B58] Wagner S. Gresser A. L. Torello A. T. Dulac C. (2006). A multireceptor genetic approach uncovers an ordered integration of VNO sensory inputs in the accessory olfactory bulb. *Neuron* 50 697–709. 10.1016/j.neuron.2006.04.033 16731509

[B59] Wei Y.-C. Wang S.-R. Jiao Z.-L. Zhang W. Lin J.-K. Li X.-Y. (2018). Medial preoptic area in mice is capable of mediating sexually dimorphic behaviors regardless of gender. *Nat. Commun.* 9:279. 10.1038/s41467-017-02648-0 29348568 PMC5773506

[B60] Wu Z. Autry A. E. Bergan J. F. Watabe-Uchida M. Dulac C. G. (2014). Galanin neurons in the medial preoptic area govern parental behaviour. *Nature* 509 325–330. 10.1038/nature13307 24828191 PMC4105201

[B61] Xu P. S. Lee D. Holy T. E. (2016). Experience-dependent plasticity drives individual differences in pheromone-sensing neurons. *Neuron* 91 878–892. 10.1016/j.neuron.2016.07.034 27537487 PMC5003430

[B62] Young J. M. Massa H. F. Hsu L. Trask B. J. (2010). Extreme variability among mammalian V1R gene families. *Genome Res.* 20 10–18. 10.1101/gr.098913.109 19952141 PMC2798821

[B63] Young J. M. Trask B. J. (2007). V2R gene families degenerated in primates, dog and cow, but expanded in opossum. *Trends Genet.* 23 212–215. 10.1016/j.tig.2007.03.004 17382427

[B64] Zhang J.-X. Liu Y.-J. Zhang J.-H. Sun L. (2008). Dual role of preputial gland secretion and its major components in sex recognition of mice. *Physiol. Behav.* 95 388–394. 10.1016/j.physbeh.2008.07.002 18657559

[B65] Zhang X. Rodriguez I. Mombaerts P. Firestein S. (2004). Odorant and vomeronasal receptor genes in two mouse genome assemblies. *Genomics* 83 802–811. 10.1016/j.ygeno.2003.10.009 15081110

