# Supplementary material for: Two Preputial Gland-Secreted Pheromones Evoke Sexually Dimorphic Neural Pathways in the Mouse Vomeronasal System
Source: Front Cell Neurosci. 2019 Oct 2;13:455. doi: 10.3389/fncel.2019.00455 (PMC6783556; doi:10.3389/fncel.2019.00455)
Supplement: Supplementary file 1 [file Data_Sheet_1.docx]

Supplementary Material

Two Preputial Gland-Secreted Pheromones Evoke Sexually Dimorphic Neural Pathways in the Mouse Vomeronasal System

**Qun Liu^1^, Xiao Guo^2^, Pan Wang^2^, Yaohua Zhang^2^, Yijun Wu^2^, Jian-Xu Zhang^2*^ and Liquan Huang^1,3*^**

**Correspondence:** Dr. Liquan Huang**:** [huangliquan@zju.edu.cn](mailto:huangliquan@zju.edu.cn),

or Dr. Jian-Xu Zhang: [zhangjx@ioz.ac.cn](mailto:zhangjx@ioz.ac.cn)

**
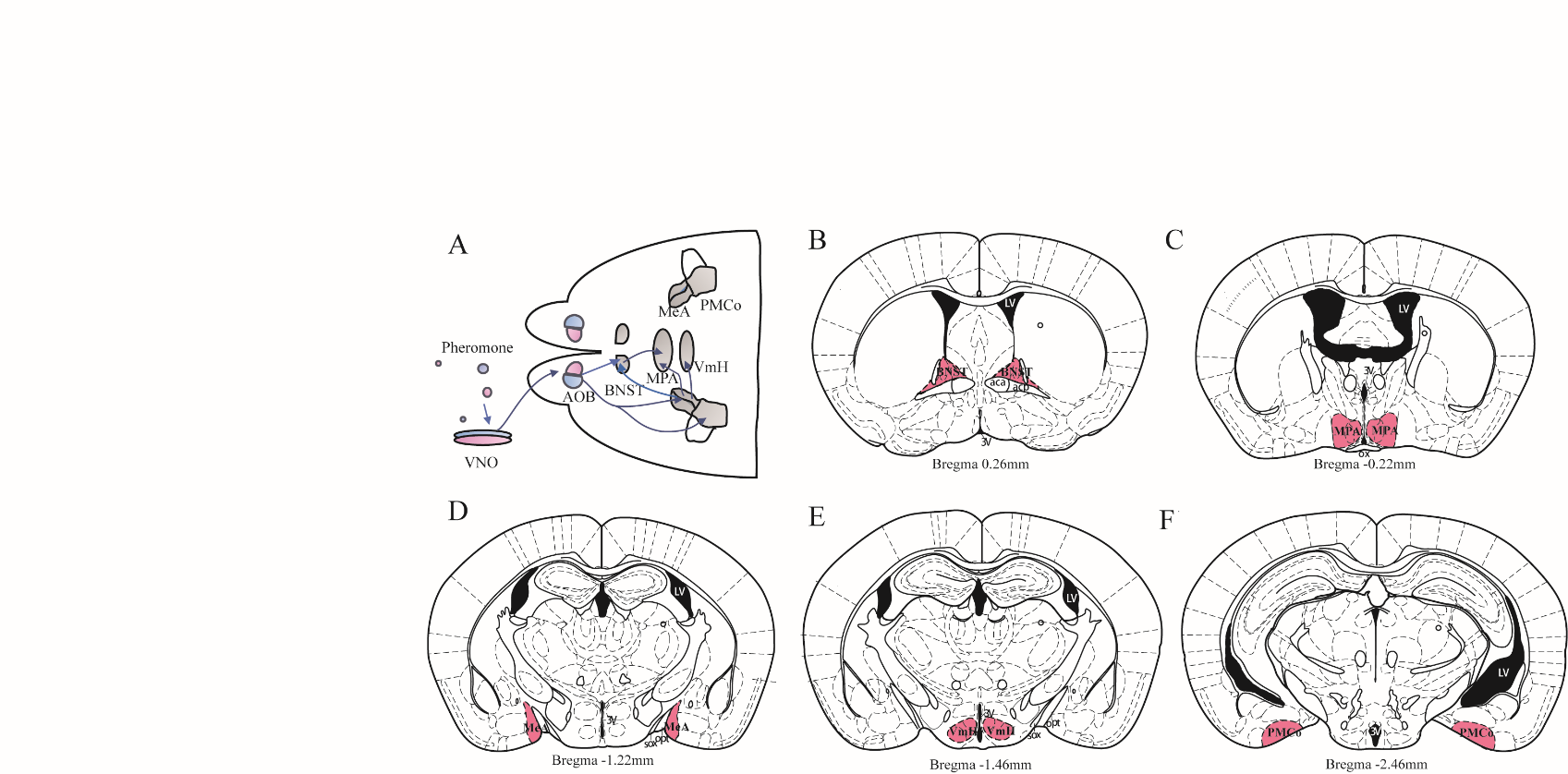
**

**Supplemental Figure 1 Diagrams of the VNO neural pathway and the brain nuclei analyzed in this study. (A)** Diagram of the VNO neural pathway: the VNO neurons project to the AOB, which in turn projects to the BNST, MeA as well as PMCo, then further down to the MPA and VmH. **(B, C, D, E and F)** Pink areas are the BNST, MPA, MeA, VmH and PMCo located at 0.26 mm in front of bregma, 0.22, 1.22, 1.46 and 2.46 mm at the back of bregma, respectively (Paxinos and Franklin, 2004). Abbreviations: VNO, vomeronasal organ; AOB, accessory olfactory bulb; BNST, bed nucleus of the stria terminalis; MPA, medial preoptic area; MeA, medial amygdaloid nucleus; VmH, ventromedial hypothalamic nucleus; PMCo, posteromedial cortical amygdaloid nucleus; LV, lateral ventricle; aca, anterior commissure, ant; acp, anterior commissure, post; 3V, 3rd ventricle; ox, optic chiasm; sox, supraoptic decussation; opt, olivary pretectal nucleus.

Paxinos, G., and Franklin, K.B.J. (2004). *The Mouse Brain in Stereotaxic Coordinates.* Elsevier Academic Press.

**Supplemental Figure 2 Quantification analysis of taste bud cells expressing TPH2 and TRPM5.** Numbers of TRPM5-positive cells and TPH2-positive cells from circumvallate, foliate and fungiform sections obtained from C57 mice. The percentage of TPH2-positive cells in the population of TRPM5-positive cells was calculated and plotted.


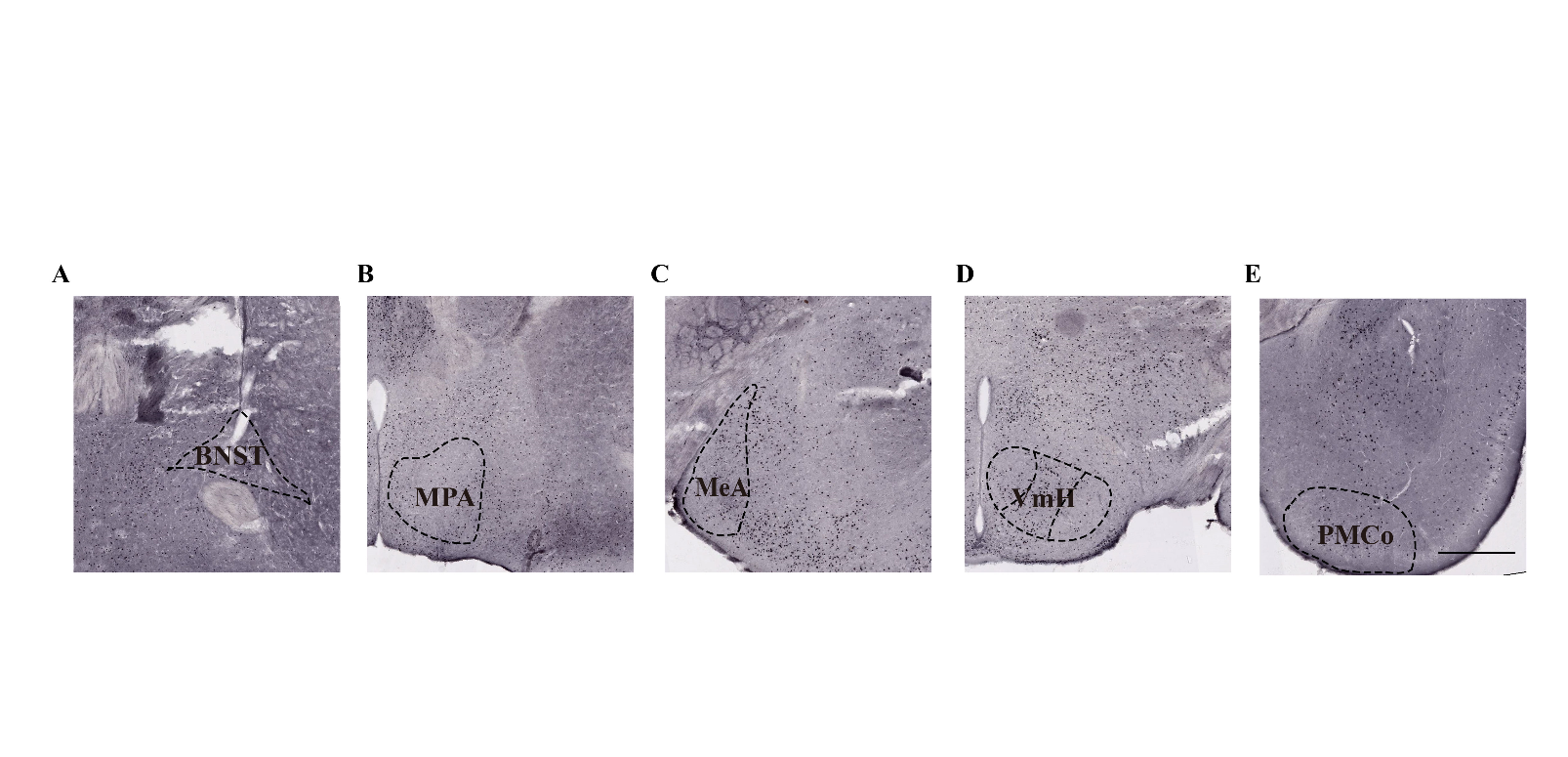


**Supplemental Figure 2 Micrographs of representative slices of mouse brain tissues through the nuclei of the VNO neural pathway. (A)** BNST; **(B)** MPA; **(C)** MeA; **(D)** VmH; **(E)** PMCo. c-Fos immunoreactive neurons in the outlined areas were counted and analyzed with the ImageJ software. Scale bar: 500 µm.
